# LMO7 Suppresses Tumor‐Associated Macrophage Phagocytosis of Tumor Cells Through Degradation of LRP1

**DOI:** 10.1002/advs.202511162

**Published:** 2025-11-09

**Authors:** Mengkai Li, Tao Wang, Yuwen Zhong, Zhiming Wang, Wei Li, Zejian Wang, Xinyi Yang, Yu Qiao, Jianfeng Xiang, Wei Gu, Zishu Wang, Lei Sun, Feng Qian

**Affiliations:** ^1^ Shanghai Frontiers Science Center of Drug Target Identification and Delivery National Key Laboratory of Innovative Immunotherapy Engineering Research Center of Cell & Therapeutic Antibody Ministry of Education School of Pharmaceutical Sciences Shanghai Jiao Tong University Shanghai 200240 P. R. China; ^2^ Department of Interventional Oncology Renji Hospital Shanghai Jiao Tong University School of Medicine Shanghai 200240 P. R. China; ^3^ Core Facility and Technical Service Center School of Pharmaceutical Sciences Shanghai Jiao Tong University Shanghai 200240 P. R. China; ^4^ Anhui Provincial Key Laboratory of Tumor Evolution and Intelligent Diagnosis and Treatment Department of Medical Oncology First Affiliated Hospital of Bengbu Medical University Bengbu Medical University Bengbu Anhui 233030 P. R. China

**Keywords:** LMO7, LRP1, phagocytosis, tumor immune microenvironment, tumor‐associated macrophage

## Abstract

Tumor‐associated macrophages (TAMs) are one of the most abundant immune cells in solid tumors and play a critical role in tumor progression. This study found that the expression of LIM domain‐only protein 7 (LMO7) in TAMs is associated with poor patient survival. LMO7 deficiency significantly inhibited tumor growth and increased the accumulation of antitumor TAMs and CD8^+^ T cells. Specifically, single‐cell RNA sequencing (scRNA‐seq) reveals that LMO7‐deficient TAMs undergo an antitumor reprogramming, characterized by upregulated expression of pro‐inflammatory and phagocytosis‐related genes. Notably, LMO7 deficiency enhances immune‐mediated tumor confinement by regulating the phagocytic activity of TAMs. Mechanistically, LMO7 inhibits TAM phagocytosis by promoting the lysine 48‐linked polyubiquitination at lysine 45 of the β chain of the phagocytic receptor low‐density lipoprotein receptor‐related protein 1 (LRP1), leading to degradation via the ubiquitin‐proteasome system. Furthermore, combined targeting of LMO7 deficiency and SIRPα blockade demonstrates synergistic antitumor efficacy. Collectively, these findings demonstrate the critical role of LMO7 in orchestrating TAM phagocytosis and suggest that LMO7 inhibition is a promising drug target to enhance cancer immunotherapy.

## Graphical abstract

LMO7 in tumor‐associated macrophages suppresses phagocytosis of tumor cells and limits cytotoxic T lymphocytes infiltration, fostering tumor progression. Mechanistically, LMO7 mediates the ubiquitination and degradation of the phagocytic receptor LRP1, impairing its ability to engulf tumor cells and driving macrophages toward an antitumor phenotype.

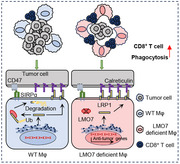

## 1 Introduction

Tumors are complex ecosystems consisting not only of highly heterogeneous cancer cells but also a diverse tumor microenvironment (TME).^[^ ^1^ ^]^ Within the TME, the tumor immune microenvironment (TIME) comprises various immune cell populations, extracellular immune factors, and cell surface molecules, all of which play crucial roles in mediating tumor–host interactions and profoundly influence tumor initiation, progression, and response to therapy.^[^ ^2^, ^3^ ^]^ Among them, tumor‐associated macrophages (TAMs) represent one of the most abundant immune cell populations and act as double‐edged swords with dual roles in cancer.^[^ ^1^, ^4^ ^]^ TAMs promote tumor progression by driving tumorigenesis and cell proliferation, enhancing invasion and migration, stimulating tumor angiogenesis, and facilitating immunosuppression.^[^ ^5^, ^6^, ^7^ ^]^ Conversely, TAM heterogeneity also enables them to exert antitumor effects by phagocytosing and eliminating tumor cells and activating adaptive immunity through antigen presentation and co‐stimulation.^[^ ^1^, ^8^, ^9^ ^]^ Thus, a deeper understanding of the multifaceted roles of TAMs in tumor development and progression could provide new insights into the TME.

Macrophage‐mediated phagocytosis is essential for maintaining TIME homeostasis and eliminating malignant cells.^[^ ^10^ ^]^ This process is tightly regulated by “eat me” signals—such as Fc receptors‐mediated signals, calreticulin‐LRP1, and SLAMF7‐MAC‐1^[^ ^8^, ^11^, ^12^, ^13^ ^]^ —and “don't eat me” signals—including CD47‐SIRPα, CD24‐Siglec‐10, MHC‐I‐LILRB1, and PD‐L1‐PD‐1.^[^ ^14^, ^15^, ^16^, ^17^ ^]^ The balance between these functional classes of molecules on cancer cells and TAMs determines the initiation of phagocytosis. For example, SIRPα, the receptor for CD47, transmits inhibitory signals that block macrophage‐mediated phagocytosis by preventing cytoskeletal rearrangement and actin polymerization.^[^ ^18^, ^19^ ^]^ In contrast, calreticulin functions as a pro‐phagocytic signal in many cancers by interacting with its receptor LRP1, thereby facilitating the recognition and engulfment of target cells.^[^ ^12^, ^20^ ^]^ Dysregulation of these pathways, such as upregulation of inhibitory signals or downregulation of phagocytic receptors, enables tumor cells to evade immune surveillance and promotes tumor progression. Targeting phagocytosis checkpoint pathways involving receptor–ligand interactions has been shown to reduce tumor growth in various preclinical models, including glioblastoma, melanoma, lymphoma, and colorectal cancer.^[^ ^17^, ^21^, ^22^, ^23^ ^]^ Therefore, elucidating the regulatory mechanisms of phagocytosis holds promise for the development of novel therapeutic strategies.

LIM domain only 7 (LMO7) is a multifunctional protein involved in cytoskeletal remodeling and epithelial integrity.^[^ ^24^, ^25^ ^]^ It has been implicated in several physiological and pathological processes, including Emery‐Dreifuss muscular dystrophy (EDMD) and wound healing.^[^ ^26^, ^27^ ^]^ Our previous study also demonstrated that LMO7 regulates fibroblasts to promote the progression of pulmonary fibrosis.^[^ ^28^ ^]^ Moreover, we highlighted the pivotal role of LMO7 in innate immune cells, particularly macrophages, where it functions as an E3 ubiquitin ligase to regulate macrophage activation and suppress inflammatory diseases.^[^ ^29^ ^]^ Recently, elevated expression of LMO7 has been observed in multiple cancer types, including colorectal, breast, liver, lung, pancreatic, gastric, and prostate cancer, suggesting its potential oncogenic role in cancer progression.^[^ ^30^, ^31^, ^32^, ^33^, ^34^ ^]^ However, the effect of LMO7 on the TIME remains to be fully elucidated.

In this study, we employed both global and myeloid‐specific conditional LMO7 knockout mice bearing subcutaneous tumors to investigate the role of LMO7 in regulating tumor immunity. Our findings reveal that LMO7 promotes tumor growth by suppressing TAM phagocytosis and impairing the recruitment of antitumor immune cells, including MHC‐II^+^ TAMs and CD8^+^ T lymphocytes. Mechanistic investigations showed that LMO7 expression in TAMs mediates the ubiquitination and subsequent degradation of LRP1, thereby inhibiting macrophage phagocytic activity. Furthermore, we developed a novel combinatorial treatment strategy and found that LMO7 deficiency synergizes with anti‐SIRPα antibody therapy to inhibit tumor growth. These findings establish LMO7 as a potential therapeutic target to enhance macrophage‐mediated cancer immunotherapy.

## 2 Results

### 2.1 LMO7 is Associated With Poor Survival in Patients and Promotes Tumor Growth in Mice

**Figure 1:**
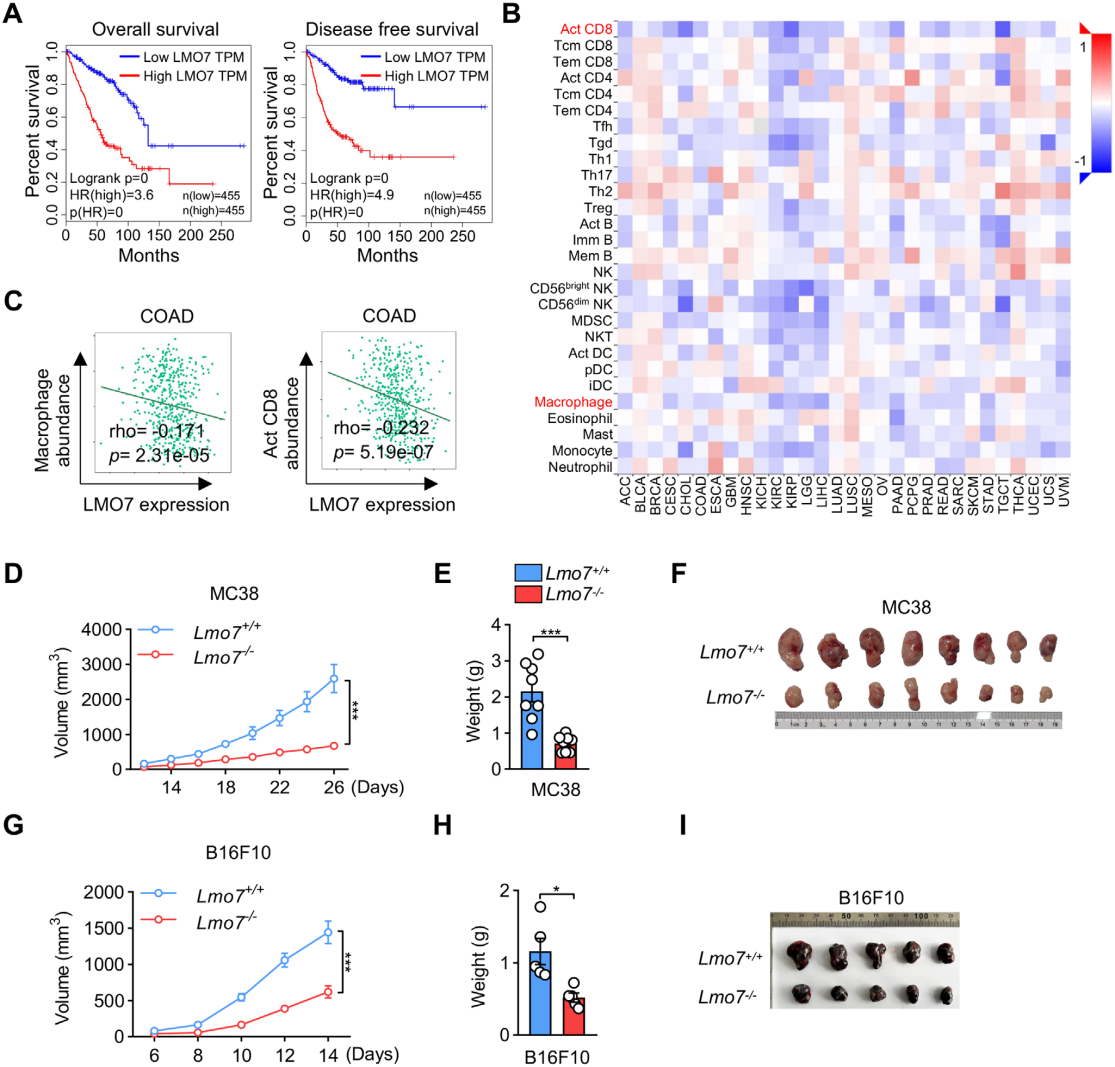
LMO7 is associated with poor survival in patients and promotes tumor growth in mice. **A)** The overall survival and disease‐free survival curves about LMO7 expression in total LUAD, COAD, and BRCA patients with quartile group cut off. Data were from GEPIA database. **B)** Heatmap of the correlation between different immune cells abundance and the LMO7 expression in various cancer patients. Data were from TISIDB database. **C)** The correlation between the LMO7 expression and macrophage or activated CD8^+^ T cells abundance in COAD. Data were from TISIDB database. Tumor growth curves **D**), tumor weight **E**), and tumor photo **F**) of MC38 bearing wild type (*Lmo7^+/+^*) and *Lmo7* knockout (*Lmo7^−/−^*) mice. Tumor growth curves **G**), tumor weight **H**), and tumor photo **I**) of B16F10 bearing *Lmo7^+/+^* and *Lmo7^−/−^* mice. The data are representative of three independent experiments (means ± SEM), *n* = 8 mice per group **D**,**E**,**F**) or *n* = 5 mice per group **G**,**H**,**I**). *p* values were analyzed using two‐way ANOVA (Sidak's test) **D**,**G**), unpaired two‐tailed *t*‐tests **E**,**H**). ^*^ *p* < 0.05, ^***^ *p* < 0.001.

Previously, we reported the regulatory effect of LMO7 in fibrotic diseases and inflammatory bowel disease.^[^ ^28^, ^29^ ^]^ However, its specific role in tumors and its involvement in the TIME remain unclear. We first investigated the clinical relevance of LMO7 expression in cancer patients. As shown in **Figure** **1**A, Kaplan‐Meier survival analysis using the GEPIA database^[^ ^35^ ^]^ revealed that high LMO7 expression was associated with unfavorable overall survival and disease‐free survival in patients with lung adenocarcinoma (LUAD), colon adenocarcinoma (COAD), and breast invasive cancer (BRCA). Additionally, analysis using the TISIDB database^[^ ^36^ ^]^ showed that LMO7 expression was negatively correlated with the abundance of macrophages and activated T cells in multiple cancer types (Figure 1B), including COAD (Figure 1C), cervical squamous cell carcinoma (CESC), kidney renal clear cell carcinoma (KIRC), pancreatic adenocarcinoma (PAAD), and stomach adenocarcinoma (STAD) (Figure S1A,B, Supporting Information). These findings underscore the pivotal role of LMO7 in tumor progression and its strong association with the tumor immune microenvironment.

To determine the significance of LMO7 in tumor development, we subcutaneously implanted colon cancer cells (MC38) or melanoma cells (B16F10) into wild‐type (*Lmo7^+/+^*) and LMO7 knockout (*Lmo7^−/−^*) mice. As shown in Figure 1D, tumors in MC38‐bearing *Lmo7^−/−^* mice exhibited significantly smaller volumes throughout the observation period compared with those in *Lmo7^+/+^* mice. At 26 days after injection, tumors were dissected, weighed and photographed. The weight and appearance of the tumors were significantly reduced in mice lacking LMO7 (Figure 1E,F). A similar decrease in tumor growth was observed in B16F10 melanoma‐bearing *Lmo7^−/−^* mice compared with *Lmo7^+/+^* controls (Figure 1G,H,I). Taken together, these results indicate that LMO7 depletion suppresses tumor progression.

### 2.2 Tumor Growth Suppression Caused by LMO7 Deficiency is Associated With Altered TIME

**Figure 2:**
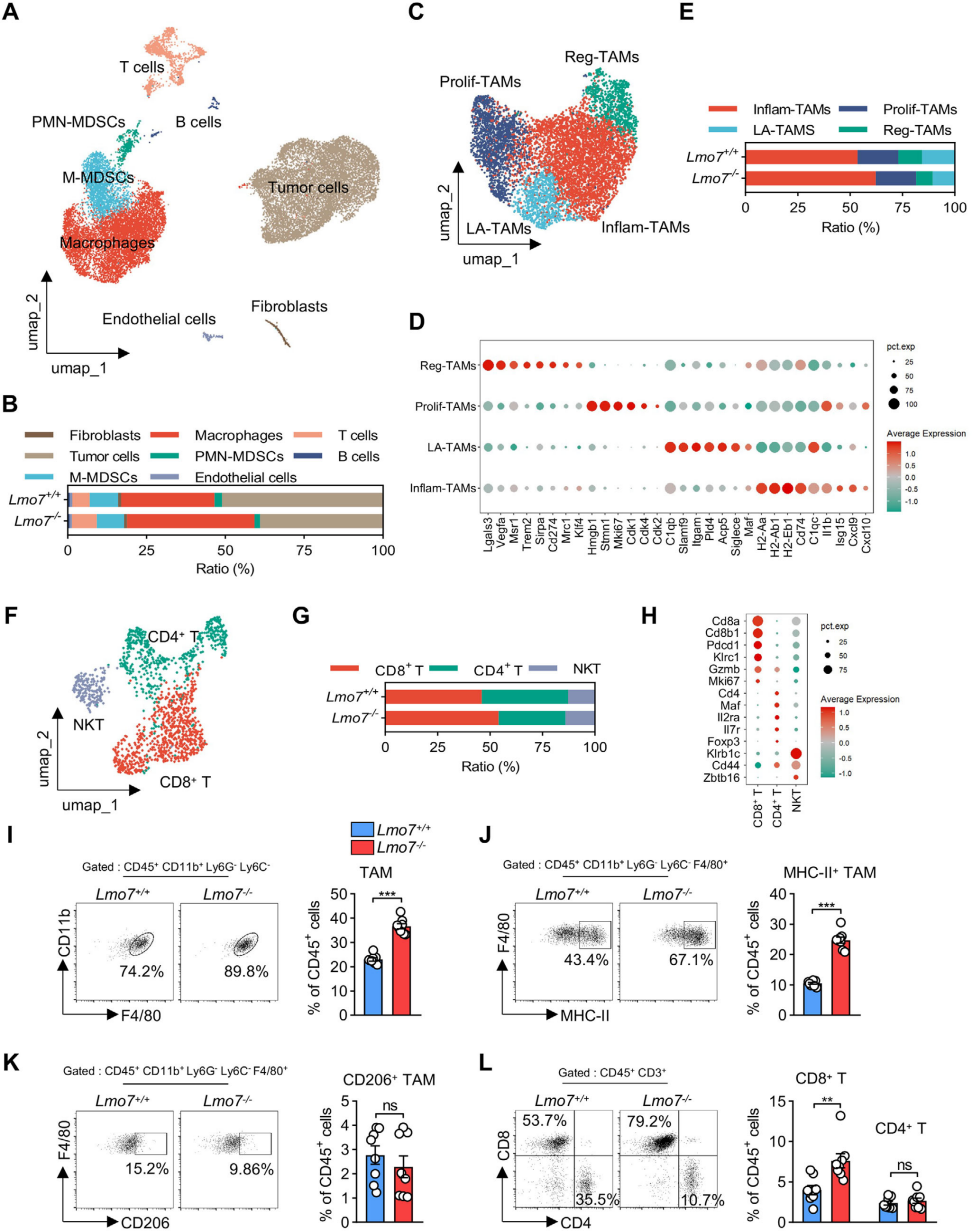
Tumor growth suppression caused by LMO7 deficiency is associated with altered TIME. **A)** Umap plot of scRNA‐seq from mice bearing MC38 tumors. **B)** Bar plot of proportional differences in cells between *Lmo7^+/+^* and *Lmo7^−/−^* mice. **C)** Umap plot displaying four types of macrophages. **D)** Dot plot presenting the expression of signature genes in four types of macrophages. **E)** Bar plot of proportional differences in various macrophages between *Lmo7^+/+^* and *Lmo7^−/−^* mice. **F)** Umap plot showing three types of T cells. **G)** Bar plot of proportional differences in various T cells between *Lmo7^+/+^* and *Lmo7^−/−^* mice. **H)** Dot plot displaying the expression of signature genes in three types of T cells. FACS analysis and quantification of tumor‐associated macrophage (TAM) (**I**), MHC‐II^+^ TAMs **J**), CD206^+^ TAMs, **K**) and T cells **L**) in MC38 tumors. The data are representative of three independent experiments (means ± SEM), n = 8 mice per group **I**,**J**,**K**,**L**). *p* values were analyzed using unpaired two‐tailed *t*‐tests **I**,**J**,**K**,**L**). ns, non‐significance, ^**^ *p* < 0.01, ^***^ *p* < 0.001.

To investigate the effects of LMO7 deficiency on the TME, we performed single‐cell RNA sequencing (scRNA‐seq) on tumor tissues from MC38 tumor‐bearing *Lmo7^+/+^* and *Lmo7^−/−^* mice. Unsupervised clustering of the single‐cell data identified 11 distinct cell clusters (Figure S2A, Supporting Information). Cell types for these clusters were annotated based on canonical cell‐type‐specific markers, which included two macrophage clusters, two epithelial cell clusters, two T cell clusters, and a single cluster of monocytic myeloid‐derived suppressor cells (M‐MDSCs), polymorphonuclear myeloid‐derived suppressor cells (PMN‐MDSCs), fibroblasts, endothelial cells, and B cells in both *Lmo7^+/+^* and *Lmo7^−/−^* mice (**Figure** **2**A; Figure S2B,C, Supporting Information). Notably, the proportion of tumor cells (malignant epithelial cells, characterized by expression of *Epcam*, *Krt14*, and *Aldh1a1*, and high copy number variation (CNV) scores) was lower in tumors from *Lmo7^−/−^* mice than in tumors from *Lmo7^+/+^* mice (Figure 2B; Figure S2C,D, Supporting Information). By contrast, the proportion of T cells (characterized by expression of *Cd3e*, *Cd3g*, and *Trac*) and macrophages (characterized by expression of *Apoe*, *C1qa*, and *Mrc1*) was higher in tumors from *Lmo7^−/−^* mice than in those from *Lmo7^+/+^* mice (Figure 2B; Figure S2C, Supporting Information). Further analysis of macrophage subsets by scRNA‐seq revealed four macrophage subtypes, including inflammation‐associated TAMs (Inflam‐TAMs, characterized by expression of MHC‐II family‐related molecules *H2‐Aa*, *H2‐Ab1*, *H2‐Eb1*, *Cd74*, cytokines *Il1b*, *Cxcl9*, *Cxcl10*, and the IFN‐regulated gene *Isg15*), immune regulatory TAMs (Reg‐TAMs, characterized by expression of *Mrc1*, *Lgals3*, *Vegfa*, *Msr1*, *Trem2*, *Sirpa*, and *Cd274*), proliferating TAMs (Prolif‐TAMs, characterized by expression of *Hmgb1*, *Stmn1*, *Mki67*, *Cdk1*, *Cdk4*, and *Cdk2*) and lipid‐associated TAMs (LA‐TAMs, characterized by expression of *Slamf9*, *Pld4*, *Acp5*, and *Maf*) (Figure 2C,D). Among these four macrophage subsets, we observed that the proportion of Inflam‐TAMs was higher in tumors from *Lmo7^−/−^* mice, whereas the proportion of Reg‐TAMs was lower compared with *Lmo7^+/+^* tumors (Figure 2E). In addition, analysis of T cell subsets by scRNA‐seq revealed that LMO7 deficiency reduced the relative proportion of CD4^+^ T cells (characterized by expression of *Cd4*, *Maf*, *Il2ra*, *Il7r*, and *Foxp3*) and increased the relative proportions of CD8^+^ T cells (characterized by expression of *Cd8a*, *Cd8b1*, *Pdcd1*, *Klrc1*, *Gzmb*, and *Mki67*), but had no significant effect on the relative proportion of natural killer T cells (NKT cells, characterized by expression of *Klrb1c*, *Cd44*, and *Zbtb16*) (Figure 2F,G,H). These data indicate that deletion of LMO7 in mice leads to marked changes in the composition of the tumor microenvironment, particularly in immune cell infiltration.

To verify the results of scRNA‐seq and evaluate the immunomodulatory effects of LMO7 deficiency on tumor development, we compared the proportions of immune cells infiltrating MC38 tumors in *Lmo7^+/+^* and *Lmo7^−/−^* mice using flow cytometry (Figure S3A, Supporting Information). In the innate immune compartment, the percentage of TAMs was ≈20% higher in *Lmo7^−/−^* mice compared with *Lmo7^+/+^* mice (Figure 2I), primarily due to an increase in MHC‐II^+^ TAMs (Figure 2J), while CD206^+^ TAMs showed no significant change (Figure 2K). The percentages of PMN‐MDSCs and M‐MDSCs were comparable between the two groups (Figure S3B, Supporting Information). In the lymphocyte compartment, *Lmo7^−/−^* mice displayed a marked increase in the proportion of CD8⁺ T cells among tumor‐infiltrating CD45⁺ immune cells (Figure 2L), while no significant differences were observed in the proportions of CD4⁺ T cells or regulatory T cells (Tregs) (Figure 2L; Figure S3C, Supporting Information). Similarly, the proportions of TAMs, MHC‐II⁺ TAMs, and CD8⁺ T cells were also notably increased in *Lmo7^−/−^* mice bearing B16F10 melanoma tumors compared with those in *Lmo7^+/+^* mice (Figure S3D–F, Supporting Information). Collectively, these findings indicate that the antitumor effects of LMO7 deficiency are driven by tumor microenvironment remodeling, characterized by increased infiltration of antitumor macrophages and CD8⁺ T cells.

### 2.3 LMO7 Deficiency Promotes Reprogramming of TAMs

**Figure 3:**
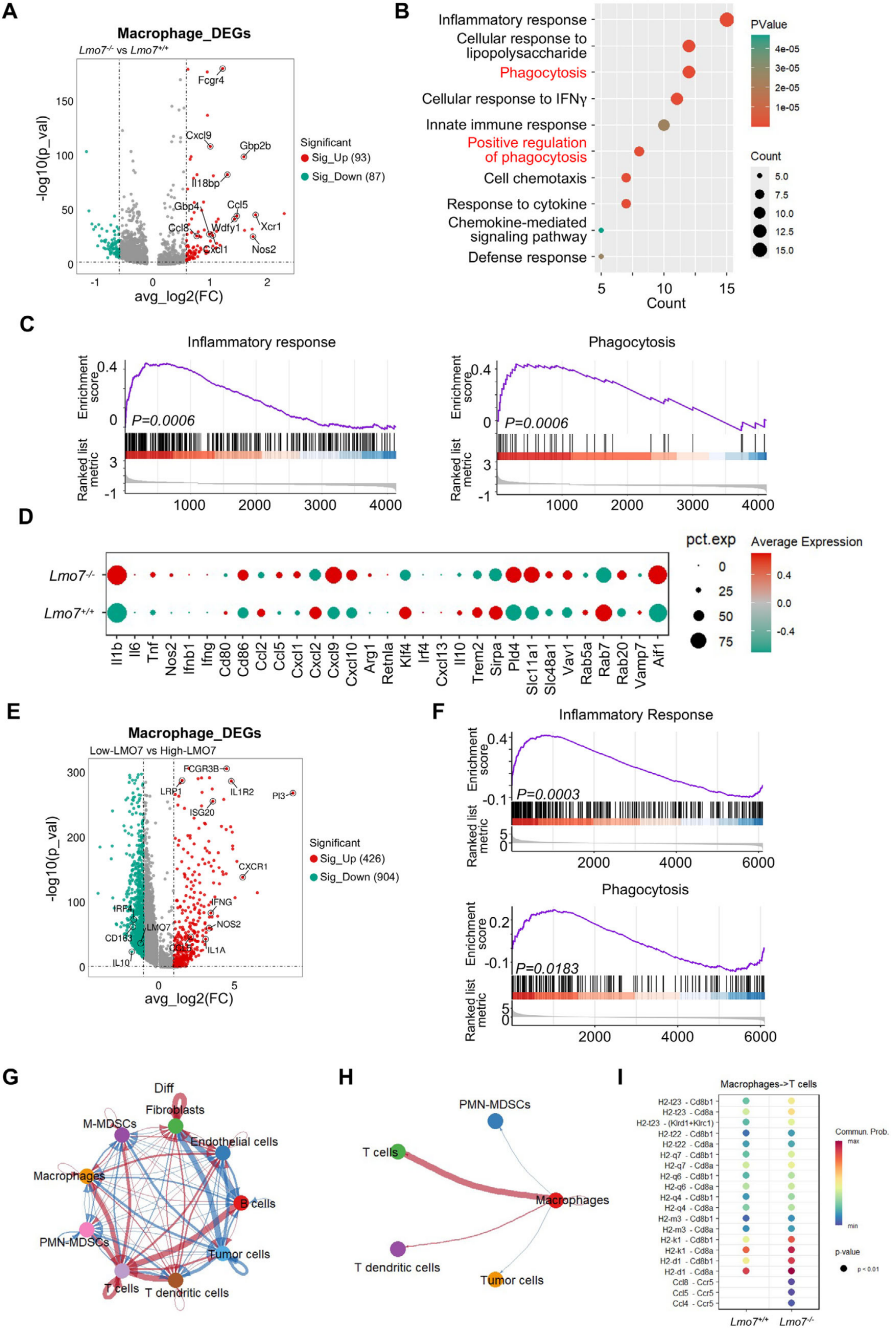
LMO7 deficiency promotes reprogramming of TAMs. **A)** Volcano plot showing the ‐log10 p_val vs log2 fold‐change of genes differentially expressed between *Lmo7^−/−^* and *Lmo7^+/+^* macrophages. **B)** GOBP enrichment analysis using the up‐regulated DEGs of macrophages. **C)** GSEA enrichment analysis using the total changed genes in macrophages. **D)** Relative expression of genes related to the function between *Lmo7^+/+^* and *Lmo7^−/−^* macrophages. **E)** Volcano plot showing the ‐log10 p_val vs log2 fold‐change of genes differentially expressed between Low‐LMO7 and High‐LMO7 macrophages from COAD patients (GSE245552). **F)** GSEA enrichment analysis using the total changed genes in Low‐LMO7 and High‐LMO7 macrophages. **G)** Network plot displaying the difference in the strength of intercellular communication between *Lmo7^−/−^* and *Lmo7^+/+^* mice (blue line represents downregulation of intercellular communication, red line represents upregulation, and the width of the line represents the strength of intercellular communication). **H)** Network plot showing the difference in the strength of intercellular communication from macrophages to others. **I)** Dot plot displaying the expression of ligand‐receptor pairs between macrophages and T cells in *Lmo7^−/−^* and *Lmo7^+/+^* mice tumor (colors represents the communication strength).

Given the significance of TAMs and T cells in the tumor immune microenvironment, we further examined which specific cell type is critically regulated by LMO7 deletion. Through scRNA‐seq profiling of TAMs, we detected 180 differentially expressed genes (DEGs) (logFC > 0.5, *p* < 0.05) between *Lmo7^−/−^* and *Lmo7^+/+^* mice, including 93 genes that were upregulated and 87 that were downregulated. Among the most significantly upregulated genes in LMO7‐deficient TAMs, many have been reported as markers of antitumor macrophages, such as *Nos2*, *Il18bp*, *Cxcl1*, *Cxcl9*, *Ccl5*, *Ccl8*, and *Fcgr4* (**Figure** **3**A). Gene Ontology Biological Process (GOBP) enrichment analysis revealed that the upregulated DEGs were mainly enriched in pathways related to inflammation and phagocytosis (Figure 3B). Gene Set Enrichment Analysis (GSEA) of all significantly changed genes (*p* < 0.05) in TAMs further confirmed increased activity in inflammatory and phagocytic pathways (Figure 3C). Additionally, antitumor‐associated and phagocytosis activation‐related genes, including *Il1b*, *Il6*, *Tnf*, *Nos2*, *Ifnb1*, *Ifng*, *Cd86*, *Ccl5*, *Cxcl1*, *Cxcl9*, *Cxcl10*, *Pld4*, *Slc11a1*, *Slc48a1*, *Vav1*, *Rab20*, and *Aif1* were more highly expressed in LMO7‐deficient macrophages. In contrast, several pro‐tumor‐associated genes, such as *Klf4*, *Irf4*, *Cxcl13*, *Il10*, *Trem2*, and *Sirpa* were expressed at lower levels in MC38 tumor‐bearing LMO7‐deficient mice than their wild type counterparts (Figure 3D). To further investigate the role of LMO7 in TAMs, we re‐analyzed scRNA‐seq data from colon adenocarcinoma patients (GSE245552).^[^ ^37^ ^]^ Based on LMO7 expression in macrophages, patient samples were divided into low‐LMO7 and high‐LMO7 groups, determined by quartiles, and subjected to integrated analysis (Figure S4A–C, Supporting Information). Differential gene expression analysis in TAMs between low‐LMO7 and high‐LMO7 groups revealed 1330 DEGs (logFC > 1, *p* < 0.05), including 426 upregulated and 904 downregulated genes (Figure 3E). The significantly upregulated genes, such as IL1A, NOS2, CCL5, IFNG, LRP1, and FCGR3B, have been reported to contribute to the killing and clearance of tumor cells (Figure 3E). GSEA of all significantly changed genes (*p* < 0.05) in TAMs indicated higher enrichment in pathways related to inflammatory responses and phagocytosis (Figure 3F; Figure S4D, Supporting Information), consistent with our experimental findings. Furthermore, macrophages with low LMO7 expression exhibited higher levels of antitumor and phagocytosis‐related genes, including IL1B, IL6, TNF, CXCL1, CXCL2, VAV1, SLC11A1, RAB20, RAB5A, SYT7, ABCA7, MYH9, CDC42, and DOCK4, whereas pro‐tumor genes, including CD163, KLF4, IRF4, and IL10, were downregulated (Figure S4E, Supporting Information). Taken together, these findings indicate that LMO7 deficiency induces an antitumor program in TAMs.

We also examined the effect of LMO7 deficiency on CD8^+^ T cells. Analysis of DEGs in CD8^+^ T cells between *Lmo7^−/−^* and *Lmo7^+/+^* mice identified 235 DEGs (logFC > 0.5, *p* < 0.05), including 73 upregulated and 162 downregulated genes (Figure S4F, Supporting Information). Cytotoxic T‐cell effector molecule genes, such as *Ifng*, *Gzmb*, *Gzmc*, *Gzme*, and, *Gzmf* were significantly upregulated in T cells in LMO7‐deficient mice (Figure S4F, Supporting Information), whereas immune checkpoint genes, including *Pdcd1* and *Ctla4*, were significantly downregulated (Figure S4F, Supporting Information). GOBP enrichment analysis showed that the upregulated DEGs were mainly enriched in pathways related to CD8^+^ T cell activation and cytotoxicity (Figure S4G, Supporting Information). Because we observed concurrent activation of T cells and macrophages, we next sought to comprehensively characterize alterations in cell–cell communication following LMO7 deficiency. We performed CellChat analysis on our scRNA‐seq dataset to map intercellular signaling networks. This analysis revealed complex cell–cell interaction networks across all cell types, with notable differences in interaction strength between *Lmo7^−/−^* mice and *Lmo7^+/+^* mice (Figure 3G). Focusing on macrophage–T cell interactions, we observed substantial alterations in communication patterns between the two groups (Figure 3H). Detailed ligand‐receptor interaction analysis between macrophages and T cells demonstrated that the MHC‐I‐CD8 signaling axis—crucial for antigen presentation and CD8^+^ T cell activation—and CCL‐CCR5 signaling axis—involved in T cell recruitment—were the most profoundly affected following LMO7 deficiency (Figure 3I). Together, these results demonstrate that LMO7 deficiency drives TAM reprogramming, enhancing their antitumor properties, including inflammation, phagocytosis, and T cell recruitment.

### 2.4 LMO7 Deficiency Enhances Immune‐Mediated Tumor Confinement via TAMs

The increased infiltration of CD8^+^ T cells and antitumor TAMs suggested that these cell types may contribute to the antitumor effect of LMO7 deficiency. T cell depletion was performed to evaluate the functional contribution of CD8^+^ T cells in the antitumor effect of LMO7 deficiency. By administering an anti‐CD3ε antibody to LMO7 knockout mice, we found that when tumor‐infiltrated CD3^+^ cells were effectively eliminated (Figure S5A, Supporting Information), both *Lmo7^+/+^* mice and *Lmo7^−/−^* mice exhibited a slight increase in tumor growth following anti‐CD3ε antibody treatment. However, T cell depletion failed to reverse the tumor suppression observed in LMO7‐deficient mice compared to control mice (Figure S5B–D, Supporting Information). Additionally, T cell depletion did not affect the increased infiltration of TAMs and MHC‐II^+^ TAMs caused by LMO7 deficiency (Figure S5E, Supporting Information). These findings indicate that the primary tumor‐suppressive effects of LMO7 deficiency are not mediated by T cells.

**Figure 4:**
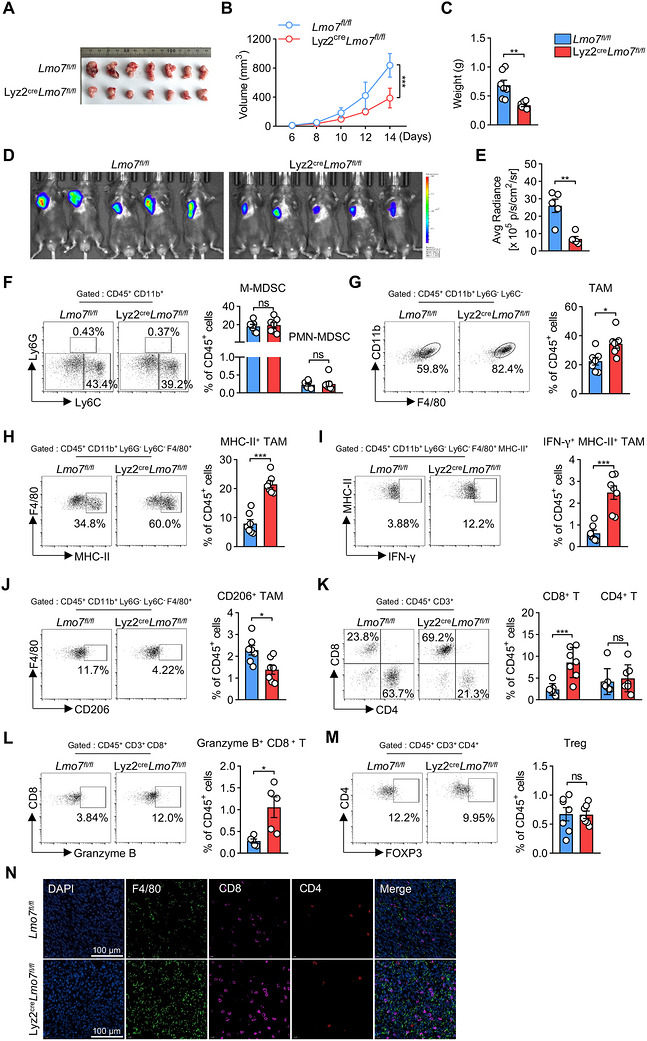
LMO7 deficiency enhances immune‐mediated tumor confinement via TAMs. Tumor photo **A**), tumor growth curves, **B**) and tumor weight **C**) of MC38 bearing *Lmo7^fl/fl^* and Lyz2^cre^ *Lmo7^fl/fl^* mice. In vivo imaging **D**) and average radiance **E**) of MC38‐luciferase bearing *Lmo7^fl/fl^* and Lyz2^cre^ *Lmo7^fl/fl^* mice. FACS analysis and quantification of tumor‐infiltrating MDSCs **F**), macrophages **G**), MHC‐II^+^ TAMs **H**), IFN‐γ^+^ MHC‐II^+^ TAMs **I**), CD206^+^ TAMs **J**), T cells **K**), granzyme B^+^ CD8^+^ T cells, **L**) and regulatory T cells **M**) in MC38 tumors. **N)** Confocal microscopy imaging of macrophages (F4/80^+^, green) and CD8^+^ T cells (violet), CD4^+^ T cells (red), and DAPI (blue) in MC38 tumor sections. Scale bars, 100 µm. The data are representative of three independent experiments (means ± SEM), n = 7 mice per group **A**–**C**,**F**–**K**,**M**) or *n* = 5 mice per group **D**,**E**,**L**). *p* values were analyzed using two‐way ANOVA (Sidak's test) **B**), unpaired two‐tailed *t*‐tests **C**,**E**–**M**). ns, non‐significance, ^*^ *p* < 0.05, ^**^ *p* < 0.01, ^***^ *p* < 0.001.

We next generated myeloid‐specific LMO7 knockout mice by crossing Lyz2^cre^ mice with *Lmo7^fl/fl^* mice to determine whether LMO7 deficiency suppresses tumor growth primarily through TAM‐mediated mechanisms (Figure S6, Supporting Information). Consistent with the results in global LMO7 knockout mice, tumor growth in Lyz2^cre^ *Lmo7^fl/fl^* mice was significantly suppressed compared to *Lmo7^fl/fl^* controls (**Figure** **4**A–C). Using MC38‐luciferase cells for in vivo imaging, we further confirmed that tumors in Lyz2^cre^ *Lmo7^fl/fl^* mice exhibited markedly reduced luminescent signals, indicating suppressed tumor growth (Figure 4D,E). Flow cytometry analysis showed that the proportions of M‐MDSCs and PMN‐MDSCs within tumor‐infiltrating CD45^+^ cells were not significantly altered by myeloid‐specific LMO7 deficiency (Figure 4F). In contrast, the proportion of TAMs increased significantly (Figure 4G), with MHC‐II⁺ TAMs representing the dominant population (Figure 4H). Notably, myeloid‐specific LMO7 deficiency also increased IFN‐γ secretion by TAMs—a cytokine associated with antitumor immune responses (Figure 4I)—and reduced the proportion of CD206⁺ TAMs, which are linked to tumor‐promoting functions (Figure 4J). Analysis of tumor‐infiltrating T lymphocytes revealed a significant increase in CD8⁺ T cells in Lyz2^cre^ *Lmo7^fl/fl^* mice (Figure 4K), accompanied by a higher proportion of CD8⁺ T cells expressing granzyme B (Figure 4L). However, no significant changes were observed in CD4⁺ T cells or regulatory T cells (Figure 4K,M). Similarly, immunofluorescence staining demonstrated elevated infiltration of F4/80⁺ macrophages and CD8⁺ T cells in the tumors of Lyz2^cre^ *Lmo7^fl/fl^* mice, whereas the infiltration of CD4⁺ T cells remained unchanged (Figure 4N). In addition, tumor growth was also suppressed in heterozygous mice (Lyz2^cre^ *Lmo7^wt/fl^*) compared to *Lmo7^wt/fl^*, with less pronounced inhibition than homozygous (33% ± 12% and 51% ± 13% growth inhibition, respectively, compared with matched controls) (Figure S7A–C, Supporting Information). And the infiltration of TAMs (Figure S7D, Supporting Information), MHC‐II^+^ TAMs (Figure S7E, Supporting Information) and CD8^+^ T cells (Figure S7G, Supporting Information) also increased in MC38 bearing heterozygous mice (Lyz2^cre^ *Lmo7^wt/fl^*), while the infiltration of CD206^+^ TAMs (Figure S7F, Supporting Information) and CD4^+^ T cells (Figure S7G, Supporting Information) remained unchanged. These findings indicate that LMO7 in macrophages dose‐dependently facilitates tumor immune escape.

Next, we assessed the effect of LMO7 in a therapeutic tumor model. In established tumor‐bearing *Lmo7^fl/f^* mice, we administered AAV9‐F4/80‐Cre to achieve macrophage‐specific LMO7 knockout post‐tumor‐engraftment. As shown in Figure S8A,B (Supporting Information), ZsGreen expression was observed in TAMs after AAV infection, and western blot analysis revealed significant LMO7 reduction in FACS‐isolated TAMs of AAV9‐F4/80‐Cre group compared to AAV9‐F4/80‐Empty group. In addition, tumor growth in *Lmo7^fl/f^* mice treated with AAV9‐F4/80‐Cre was significantly smaller than those in control group (Figure S8C–E, Supporting Information). Moreover, inducible ablation of macrophage LMO7 increased the proportions of TAMs, MHC‐II⁺ TAMs, and CD8^+^ T cells (Figure S8F,G,I, Supporting Information), while the proportions of CD206^+^ TAMs and CD4^+^ T cells remained unchanged (Figure S8H,I, Supporting Information). Taken together, our results demonstrate that macrophage‐specific LMO7 deficiency exerted therapeutic effects, achieved not only by improving TAM functionality but also through promoting CD8⁺ T cell recruitment and activation.

### 2.5 LMO7 Deficiency Enhances TAM Phagocytosis of Tumor Cells

**Figure 5:**
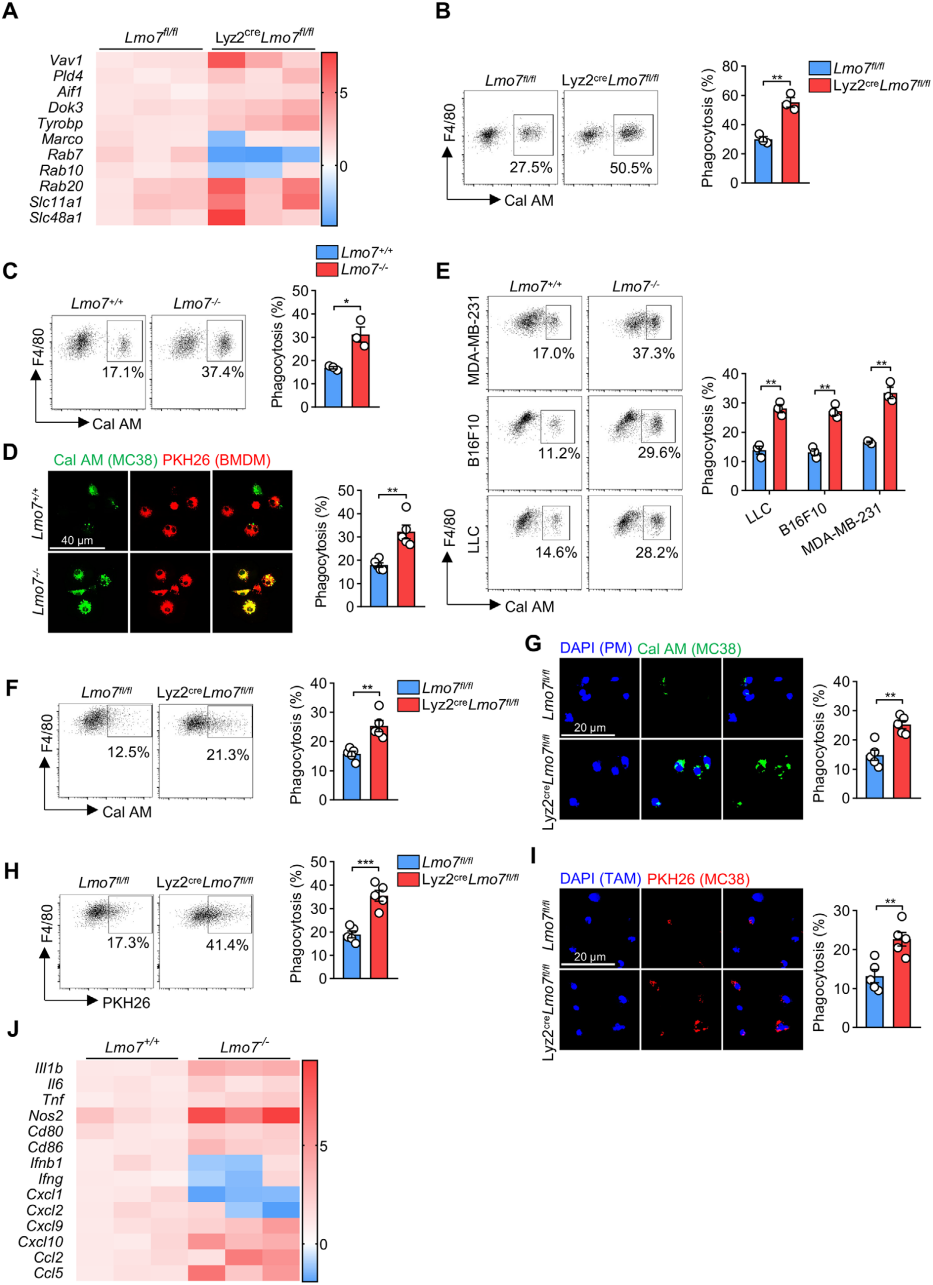
LMO7 deficiency enhances TAM phagocytosis of tumor cells. **A)** Heatmap showing normalized expression of phagocytosis‐related genes between FACS‐sorted TAMs from MC38 tumors in *Lmo7^fl/fl^* and Lyz2^cre^ *Lmo7^fl/fl^* mice. **B)** FACS analysis and quantification of phagocytic activity by *ex vivo* sorted TAMs toward Cal AM‐labeled MC38 tumor cells with anti‐CD47 antibody treatment. **C)** FACS analysis and quantification of phagocytic activity in wild type versus *Lmo7* knockout BMDMs toward Cal AM‐labeled MC38 tumor cells with anti‐CD47 antibody treatment. **D)** Confocal microscopy imaging of PKH26‐stained BMDMs engulfing Cal AM‐labeled MC38 tumor cells with anti‐CD47 antibody treatment. Scale bars, 40 µm. **E)** FACS analysis and quantification of phagocytic activity in wild type versus *Lmo7* knockout BMDMs toward Cal AM‐labeled LLC, B16F10, and MDA‐MB‐231 tumor cells with anti‐CD47 antibody treatment. **F)** FACS analysis and quantification of phagocytic activity in PMs toward Cal AM‐labeled MC38 tumor cells with anti‐CD47 antibody treatment in vivo. **G)** Confocal microscopy imaging of FACS‐isolated PMs (stained by DAPI) engulfing Cal AM‐labeled MC38 tumor cells with anti‐CD47 antibody treatment. Scale bars, 20 µm. **H)** FACS analysis and quantification of phagocytic activity in TAMs toward PKH26‐labeled MC38 tumor cells in vivo. **I)** Confocal microscopy imaging of FACS‐sorted TAMs (stained by DAPI) engulfing PKH26‐labeled MC38 tumor cells. Scale bars, 20 µm. **J)** Heatmap of relative gene expression in *Lmo7* ^−/−^ and *Lmo7* ^+/+^ BMDMs following phagocytosis of MC38 cells. The data are representative of three independent experiments (means ± SEM), *n* = 3 mice per group **A**,**B**) or *n* = 5 mice per group **F**,**H**) or *n* = 5 independent fields **D**,**G**,**I**). *p* values were analyzed using unpaired two‐tailed *t*‐tests **B**–**I**). ^*^ *p* < 0.05, ^**^ *p* < 0.01, ^***^ *p* < 0.001.

Given the observed upregulation of phagocytosis‐related pathways in LMO7‐deficient TAMs (Figure 3B–F), we sought to assess the effect of LMO7 on TAM phagocytic activity. We first measured the expression of phagocytosis‐related genes in FACS‐isolated TAMs (CD45^+^CD11b^+^Ly6G^−^Ly6C^−^F4/80^+^) from MC38‐bearing *Lmo7^fl/fl^* and Lyz2^cre^ *Lmo7^fl/fl^* mice. The results showed that phagocytosis‐related genes such as *Vav1*, *Pld4*, *Dok3*, *Tyrobp*, *Rab20*, *Slc11a1*, and *Slc48a1* were significantly upregulated in LMO7‐deficient TAMs compared with those in control TAMs (**Figure** **5**A). Consistently, when these TAMs were co‐cultured with MC38 cells to assess phagocytosis, TAMs sorted from MC38‐bearing Lyz2^cre^ *Lmo7^fl/fl^* mice exhibited enhanced phagocytosis of tumor cells compared with those from *Lmo7^fl/fl^* mice (Figure 5B), supporting our hypothesis that LMO7 deficiency promotes macrophage phagocytosis of tumor cells.

To further validate these findings, we performed in vitro phagocytosis assays by co‐culturing MC38 cancer cells with *Lmo7^−/−^* and *Lmo7^+/+^* macrophages. After treatment with anti‐CD47 antibody to block the CD47‐SIRPα inhibitory axis, LMO7‐deficient macrophages exhibited an ≈20% increase in phagocytosis of tumor cells compared with wild‐type macrophages (Figure 5C). Confocal laser microscopy confirmed that LMO7‐deficient macrophages showed enhanced engulfment of tumor cells (Figure 5D). We also extended this analysis to other tumor types, including Lewis lung cancer (LLC), melanoma (B16F10), and breast cancer (MDA‐MB‐231) cells. LMO7‐deficient macrophages consistently displayed increased phagocytic activity across all tested cancer cell lines following anti‐CD47 antibody treatment (Figure 5E). In addition, we assessed macrophage phagocytosis in vivo. MC38 cells pre‐treated with anti‐CD47 antibodies were injected into the peritoneal cavity of mice. Peritoneal macrophages (PMs) were then isolated and analyzed for phagocytic activity. Macrophages from Lyz2^cre^ *Lmo7^fl/fl^* mice exhibited significantly enhanced phagocytosis compared with those from *Lmo7^fl/fl^* mice (Figure 5F). Confocal laser microscopy further confirmed that FACS‐isolated LMO7‐deficient PMs showed enhanced engulfment of tumor cells (Figure 5G). To examine macrophage phagocytosis at tumor sites, we inoculated *Lmo7^fl/fl^* and Lyz2^cre^ *Lmo7^fl/fl^* mice with PKH26‐labeled MC38 cells and then examined PKH26‐labeled TAMs. The results showed that LMO7‐deficient TAMs were more effective at directly phagocytosing tumor cells (Figure 5H), and confocal laser microscopy further confirmed that FACS‐sorted LMO7‐deficient TAMs displayed enhanced phagocytosis of tumor cells (Figure 5I).

Macrophages that engulf tumor cells typically exhibit a pro‐inflammatory phenotype.^[^ ^38^ ^]^ To explore whether macrophage LMO7 deficiency affects this phenotype, we measured inflammation‐related genes expression in *Lmo7^−/−^* and *Lmo7^+/+^* BMDMs following MC38 cell phagocytosis. Comparing with wild‐type controls, LMO7‐deficient macrophages exhibited increased expression of pro‐inflammatory cytokines and chemokines, including *Il1b*, *Tnfa*, *Nos2*, *Cxcl9*, *Cxcl10*, *Ccl2*, and *Ccl5* (Figure 5J). Altogether, these results suggest that LMO7 deficiency enhances TAM‐mediated phagocytosis of tumor cells, contributing to an antitumor immune microenvironment that supports tumor suppression.

### 2.6 LMO7 Deficiency Enhances Macrophage Phagocytosis Through LRP1

**Figure 6:**
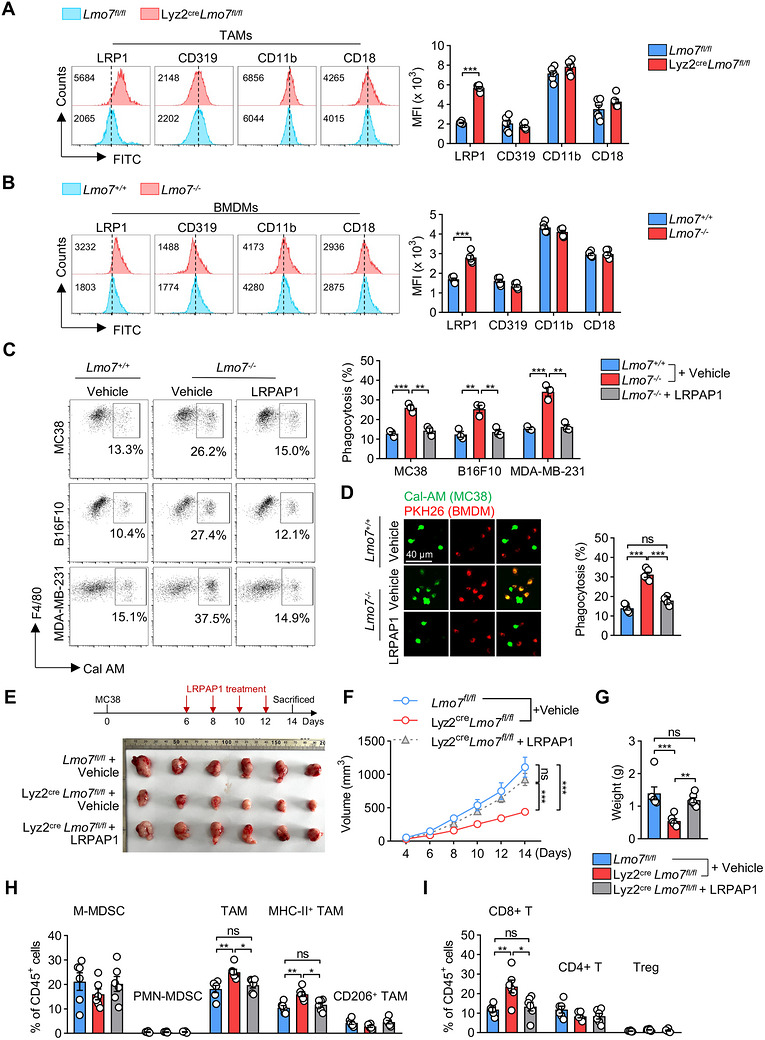
LMO7 deficiency enhances macrophage phagocytosis through LRP1. Mean fluorescence intensity (MFI) and quantification of phagocytic receptor LRP1, CD319, CD11b, and CD18 expression on TAMs **A**) and BMDMs **B**). **C)** FACS analysis and quantification of phagocytic activity in wild type versus *Lmo7* knockout BMDMs toward Cal AM‐labeled MC38, B16F10, and MDA‐MB‐231 tumor cells, with or without LRPAP1 treatment. **D)** Confocal microscopy imaging of PKH26‐stained BMDMs engulfing Cal AM‐labeled MC38 tumor cells, with or without LRPAP1 treatment. Scale bars, 40 µm. MC38 bearing Lyz2^cre^ *Lmo7^fl/fl^* mice were treated with LRPAP1 on days 6, 8, 10, and 12. Tumor photo **E**), tumor growth curves **F**), and tumor weight **G**) of MC38 bearing *Lmo7^fl/fl^* and Lyz2^cre^ *Lmo7^fl/fl^* mice. FACS analysis and quantification of tumor‐infiltrating MDSCs, macrophages **H**), and T cells **I**). The data are representative of three independent experiments (means ± SEM), *n* = 6 mice per group **A**,**B**,**E**–**I**) or *n* = 5 independent fields **D**). *p* values were analyzed using unpaired two‐tailed *t*‐tests **A**,**B**), one‐way ANOVA (Tukey's test) **C**,**D,G,H, I**), two‐way ANOVA (Sidak's test) **F**). ns, non‐significance, ^*^ *p* < 0.05, ^**^ *p* < 0.01, ^***^ *p* < 0.001.

To investigate the mechanisms by which LMO7 modulates macrophage phagocytosis, we analyzed the expression of key phagocytic receptors, including LRP1, CD319, CD11b, and CD18 on TAMs and BMDMs. Flow cytometry revealed that LRP1 expression was significantly increased in both LMO7‐deficient TAMs and BMDMs, whereas the expression levels of other receptors remained unchanged (**Figure** **6**A,B). To determine whether enhanced phagocytic activity of LMO7‐deficient macrophages was dependent on LRP1, we used LRPAP1, a specific LRP1 inhibitor, in phagocytosis assays. Blocking LRP1 significantly reduced the phagocytic activity of LMO7‐deficient macrophages against various cancer cell lines, including MC38, B16F10, and MDA‐MB‐231 cells (Figure 6C). Confocal imaging confirmed that LRP1 inhibition abolished the enhanced engulfment observed in LMO7‐deficient macrophages (Figure 6D). To investigate the role of LRP1‐dependent phagocytosis in tumor suppression resulting from conditional LMO7 deletion in macrophages, we performed in vivo functional analysis. Tumor‐bearing mice received intratumoral injections of LRPAP1 on days 6, 8, 10, and 12 after MC38 cell implantation. LRPAP1 treatment abrogated the tumor suppression observed in Lyz2^cre^ *Lmo7^fl/fl^* mice, leading to tumor growth comparable to that of *Lmo7^fl/fl^* mice (Figure 6E–G). Flow cytometry analysis of immune cells revealed that LRP1 blockade reversed the increased infiltration of TAMs, MHC‐II^+^ TAMs, and CD8^+^ T cells caused by LMO7 deficiency (Figure 6H,I). Collectively, these findings indicate that LMO7 deficiency enhances macrophage phagocytosis by upregulating LRP1 expression and that LRP1 plays a central role in mediating the antitumor effects of LMO7‐deficient macrophages.

### 2.7 LMO7 Interacts With and Promotes Degradation of LRP1 β Chain

**Figure 7:**
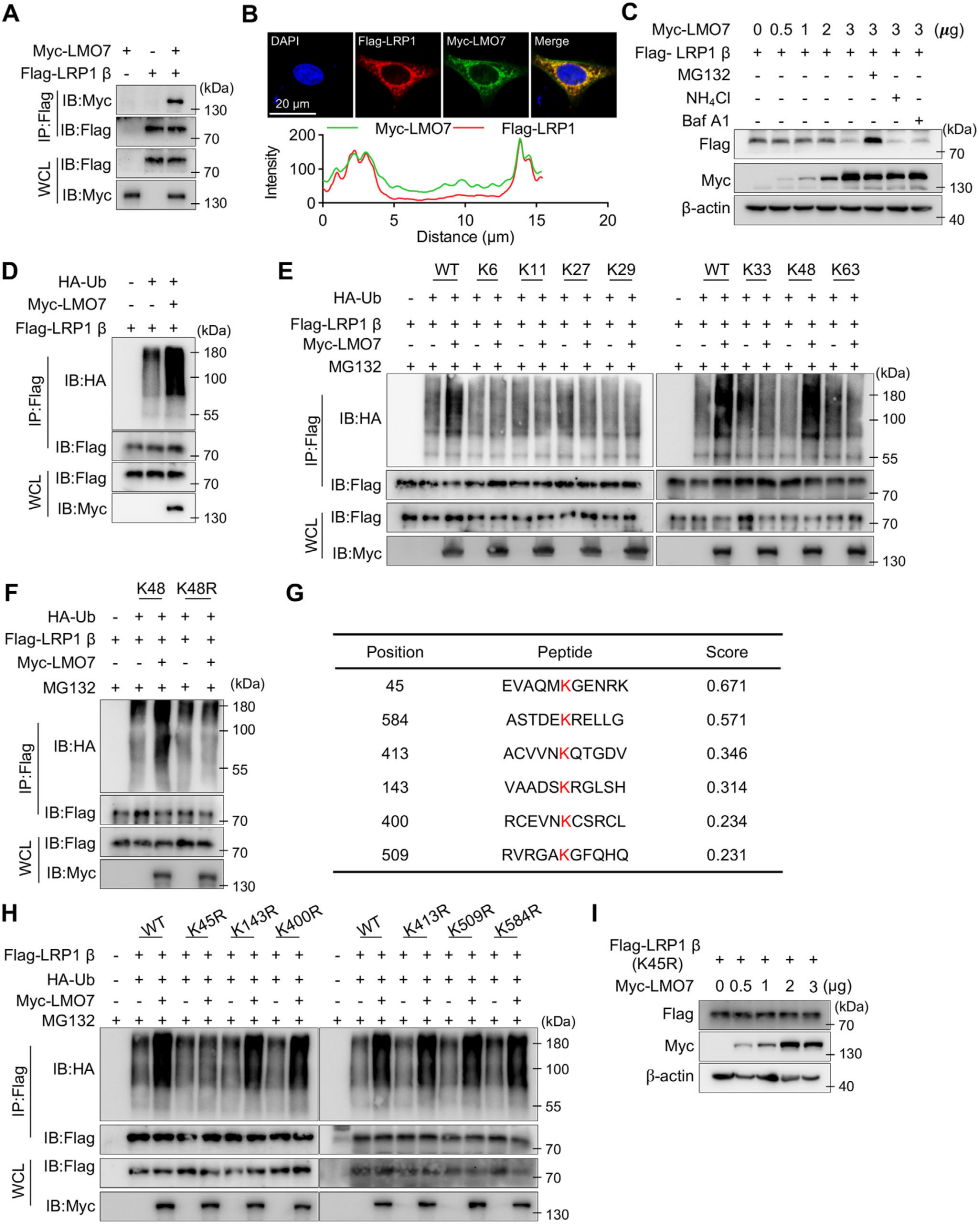
LMO7 interacts with and promotes degradation of LRP1 β chain. **A)** Co‐immunoprecipitation and immunoblot analysis of HEK293T cells co‐transfected with Flag‐LRP1 β chain and Myc‐LMO7, followed by IP with the anti‐Flag antibody. WCL, whole‐cell lysate. **B)** Confocal microscopy imaging of HeLa cells expressing Flag‐LRP1 β chain (red) and Myc‐LMO7 (green) with antibody to the appropriate protein, along with DAPI (blue). Scale bars, 20 µm. Bottom: Pearson correlation analysis of fluorescence intensities. **C)** Immunoblot (IB) analysis of LRP1 β chain protein expression in HEK293T cells transfected with Flag‐LRP1 β chain and different amounts of Myc‐LMO7. MG132, NH4Cl or Bafilomycin A1 were added to treat the cells for 6 h, if necessary. β‐actin served as a loading control. **D)** Immunoprecipitation (IP) and immunoblot (IB) analysis of ubiquitination of LRP1 β chain in HEK293T cells co‐transfected with Flag‐LRP1 β chain, HA‐Ub, along with Myc‐LMO7. **E,F)** HEK293T cells were transfected with Flag‐LRP1 β chain and Myc‐LMO7, along with HA‐Ub or its mutants (K6, K11, K27, K29, K33, K48, K63, and K48R). Cell lysates were subjected to immunoprecipitation by the anti‐Flag antibody, and then immunoblotted with the indicated antibodies. **G)** The potential ubiquitination sites on LRP1 β chain predicted by GPS‐Uber database. **H)** Flag‐LRP1 β chain or its mutants (K45R, K143R, K400R, K413R, K509R, and K584R) were individually transfected into HEK293T cells, along with Myc‐LMO7 and HA‐Ub. Cell lysates were subjected to immunoprecipitation by the anti‐Flag antibody, and then immunoblotted with the indicated antibodies. **I)** Immunoblot analysis of LRP1 β chain levels in HEK293T cells transfected with Flag‐LRP1 β chain (K45R) mutant with different amounts of Myc‐LMO7. β‐actin acted as a loading control. The data are representative of three independent experiments.

To investigate how LMO7 regulates the expression of LRP1, we first examined whether LMO7 physically interacts with LRP1. LRP1 is a heterodimeric receptor composed of an extracellular α chain and a transmembrane β chain, with the β chain undergoing endocytosis during receptor recycling or activation.^[^ ^39^, ^40^ ^]^ Co‐immunoprecipitation assays following overexpression of Myc‐LMO7 and Flag‐LRP1 β chain in HEK293T cells demonstrated an interaction between LMO7 and LRP1 β chain (**Figure** **7**A). Immunofluorescence imaging confirmed their colocalization in the cytoplasm of HeLa cells (Figure 7B). These results indicate that LMO7 can interact with LRP1 through LRP1 β chain.

Since LMO7 functions as an E3 ubiquitin ligase, we next tested whether it regulates LRP1 protein levels. Immunoblot analysis showed that LMO7 overexpression significantly reduced the protein levels of LRP1 β chain (Figure 7C). Furthermore, treatment with the proteasome inhibitor MG132 restored LRP1 β chain expression, whereas lysosome inhibitors (Bafilomycin A1 and NH4Cl) had no effect, supporting a proteasome‐dependent degradation mechanism (Figure 7C). To determine whether LMO7 promotes ubiquitination of the LRP1 β chain, we co‐transfected HEK293T cells with Myc‐LMO7, Flag‐LRP1 β chain, and HA‐tagged ubiquitin (HA‐Ub) plasmids. Overexpression of Myc‐LMO7 markedly enhanced polyubiquitination of the LRP1 β chain in the presence of MG132 (Figure 7D). Ubiquitin chains can form through various lysine linkages, including Lys6, Lys11, Lys27, Lys29, Lys33, Lys48, and Lys63. To identify the specific ubiquitin linkage type, we employed mutant ubiquitin constructs that retain only one lysine residue (K6, K11, K27, K29, K33, K48, or K63) while replacing all others with arginine. The results showed that LMO7 predominantly mediates K48‐linked polyubiquitination of the LRP1 β chain (Figure 7E). This was further confirmed by using the K48R Ub mutant, in which lysine 48 is substituted with arginine, thereby abolishing LMO7‐induced LRP1 β chain ubiquitination (Figure 7F). These findings suggest that LMO7 promotes K48‐linked polyubiquitination of the LRP1 β chain.

To identify the precise ubiquitination site targeted by LMO7, we used GPS‐Uber, a bioinformatics prediction tool, to assess potential lysine residues on the LRP1 β chain.^[^ ^41^ ^]^ Several candidates were identified, including Lys45, Lys143, Lys400, Lys413, Lys509, and Lys584 (Figure 7G). These residues were individually mutated to arginine to generate single‐point mutants (K45R, K584R, K413R, K143R, K400R, K509R). Among these, mutation of Lys45 to arginine (K45R) nearly abolished LMO7‐induced polyubiquitination of the LRP1 β chain (Figure 7H). Moreover, overexpression of LMO7 failed to reduce LRP1 β chain protein level in the K45R mutant, confirming the functional relevance of this site (Figure 7I). In conclusion, these results demonstrate that LMO7 interacts with the LRP1 β chain and promotes its degradation via K48‐linked polyubiquitination at Lys45 on the LRP1 β chain, thereby reducing LRP1 surface expression and impairing macrophage phagocytic activity.

### 2.8 The Combination of LMO7 Inhibition and Anti‐SIRPα Antibody Amplifies TAM‐Mediated Tumor Regression

**Figure 8:**
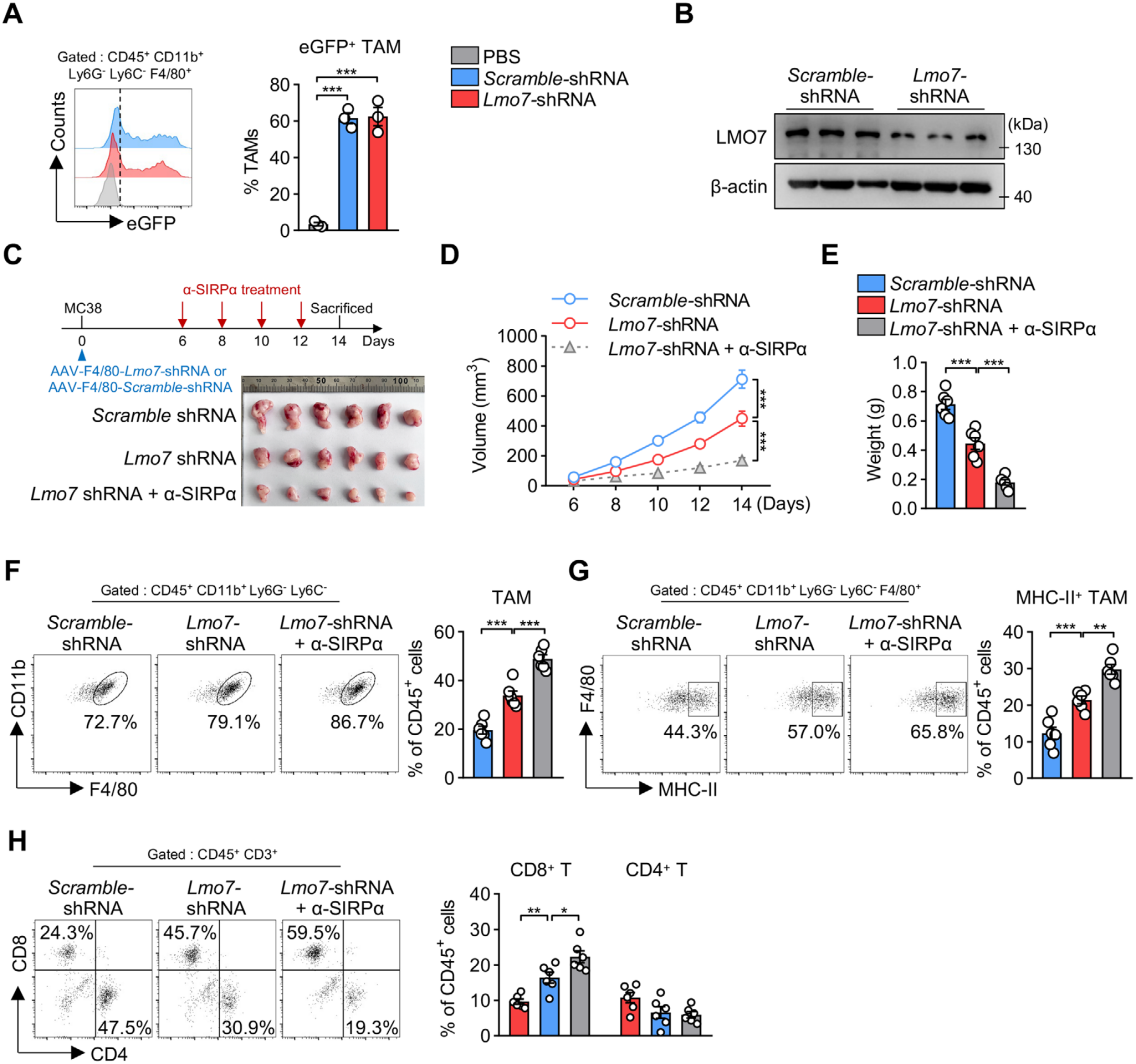
Therapeutic AAV‐mediated LMO7 silencing synergizes with SIRPα blockade to potentiate tumor regression. MC38 bearing wild type mice were treated with AAV9‐F4/80‐*Scramble*‐shRNA or AAV9‐F4/80‐*Lmo7*‐shRNA virus via tail vein on day 0 and treated with anti‐SIRPα antibody on days 6, 8, 10, and 12. **A)** FACS analysis and quantification of the eGFP fluorescence ratio of TAMs. **B)** The expression of LMO7 in FACS‐isolated TAMs. Tumor photo **C**), tumor growth curves **D**), and tumor weight **E**) of MC38 bearing wild type mice. FACS analysis and quantification of TAMs **F**), MHC‐II^+^ TAMs, **G**) and T cells **H**). The data are representative of three independent experiments (means ± SEM), n = 3 mice per group (**A**, **B**), *n* = 6 mice per group **C**–**H**). *p* values were analyzed using two‐way ANOVA (Sidak's test) **C**), one‐way ANOVA (Tukey's test) **A**,**E**–**H**). ns, non‐significance, ^*^ *p* < 0.05, ^**^ *p* < 0.01, ^***^ *p* < 0.001.

Despite the therapeutic promise of phagocytosis checkpoint blockade, two fundamental constraints persist: i) dose‐limiting hematopoietic toxicity of CD47‐targeting agents, and ii) the marginal single‐agent activity of SIRPα inhibitors. Our discovery that LMO7 acts as a tunable regulator of macrophage “eat‐me” signaling, motivates its therapeutic exploitation in parallel with “don't‐eat‐me” pathway inhibition. We first evaluated the effect of combined SIRPα antibody in macrophage‐specific LMO7‐deficient mice. Our results showed limited antitumor efficacy of anti‐SIRPα monotherapy, whereas the combination group (LMO7‐deficient mice + anti‐SIRPα) exhibited significantly enhanced tumor suppression (Figure S9A–C, Supporting Information). In addition, combination therapy also promoted the infiltration of TAMs (Figure S9D, Supporting Information), MHC‐II^+^ TAMs (Figure S9E, Supporting Information), and CD8^+^ T cells (Figure S9F, Supporting Information), while the proportion of CD4^+^ T cells remained unchanged (Figure S9F, Supporting Information). Subsequently, to develop a therapeutically translatable strategy beyond knockout models, we employed AAV‐mediated LMO7 silencing (AAV9‐F4/80‐*Lmo7‐*shRNA) coupled with anti‐SIRPα antibody treatment to evaluate combinatorial antitumor efficacy in established syngeneic tumors. As shown in **Figure** **8**A,B, eGFP expression was observed in TAMs after AAV infection, and western blot analysis revealed LMO7 reduction in FACS‐isolated TAMs of AAV9‐F4/80‐*Lmo7‐*shRNA group compared to AAV9‐F4/80‐*Scramble‐*shRNA group. AAV9‐F4/80‐*Lmo7‐*shRNA monotherapy achieved 38% ± 6% tumor growth inhibition, while the combination with anti‐SIRPα enhanced suppression to 75% ± 6% (Figure 8C–E). In addition, this combination therapy also improved the tumor immune microenvironment, which further promoted the infiltration of TAMs (Figure 8F), MHC‐II+ TAMs (Figure 8G), and CD8+ T cells (Figure 8H). Collectively, these data establish that combinatorial targeting of LMO7 deficiency and SIRPα blockade synergistically enhances macrophage‐mediated antitumor immunity, providing a novel therapeutic paradigm for overcoming current limitations in phagocytosis checkpoint immunotherapy.

## 3 Discussion

Tumor‐associated macrophages have emerged as important targets in cancer immunotherapy due to their potent phagocytic activity and their regulatory role in inflammation.^[^ ^1^, ^42^ ^]^ In this study, we identified LMO7 as a negative regulator of TAM‐mediated phagocytosis. LMO7 deficiency in macrophages significantly suppressed tumor growth by enhancing tumor cell engulfment, promoting the expression of antitumor genes, and increasing CD8^+^ T cell infiltration. Mechanistically, LMO7 catalyzed the ubiquitination and proteasomal degradation of LRP1, thereby restricting macrophage phagocytic function. The combined treatment of LMO7 deficiency and anti‐SIRPα antibody further inhibited tumor progression. These findings reveal a previously unrecognized role of LMO7 in regulating TAM phagocytosis and propose LMO7 as a potential target for immunotherapy.

TAMs represent a major component of the TIME and exhibit robust phagocytic capacity.^[^ ^43^ ^]^ Increasing evidence suggests that macrophage phagocytosis contributes to tumor suppression through multiple mechanisms.^[^ ^44^ ^]^ First, direct phagocytosis of tumor cells by TAMs can restrict tumor growth.^[^ ^38^, ^45^ ^]^ Second, TAMs act as antigen presenting cells, priming effector T cell responses against tumor cells.^[^ ^46^, ^47^, ^48^ ^]^ Third, phagocytosis modulates TAM polarization, promoting the secretion of tumor‐suppressive cytokines and enhancing antitumor immunity.^[^ ^38^, ^49^ ^]^ In our study, LMO7 deletion led to tumor suppression accompanied by increased infiltration of TAMs and CD8⁺ T cells. Importantly, the antitumor effects of LMO7 deficiency were primarily attributed to its regulation of macrophage function rather than direct effects on T cells. While our previous work has implicated LMO7 in controlling inflammatory cytokine production in macrophages,^[^ ^29^ ^]^ we now demonstrate its non‐redundant role in controlling phagocytic capacity and immune regulation. Notably, pharmacological inhibition of LRP1 with LRPAP1 abrogated the enhanced phagocytosis and CD8^+^ T cell infiltration observed in LMO7‐deficient macrophages, further supporting the critical role of macrophage phagocytosis in mediating the antitumor effects of LMO7 deletion.

LRP1 is a multifunctional endocytic and signaling receptor belonging to the LDL receptor (LDLR) gene family and is widely expressed across various tissues.^[^ ^39^ ^]^ LRP1 has been postulated to participate in numerous diverse physiological and pathological processes ranging from plasma lipoprotein homeostasis, atherosclerosis, and fibrinolysis to neuronal regeneration and survival.^[^ ^50^, ^51^, ^52^ ^]^ In vivo studies have shown that LRP1 contributes to cancer progression through its roles in tumor cell proliferation, migration, and invasion.^[^ ^53^, ^54^, ^55^ ^]^ In the context of the tumor microenvironment, LRP1 in macrophages serves not only as a receptor for “eat me” signals but also as a modulator of inflammatory responses. For example, it was reported that monocytes infiltration was strongly enhanced in PanO2 pancreatic carcinoma cells‐bearing myeloid cells specific LRP1‐deficient mice, and it also showed that LRP1‐deficient macrophages expressed high levels of CCL3 and vascular endothelial growth factor (VEGF) into the tumor microenvironment to regulate the progression of the tumor.^[^ ^56^ ^]^ Thus, targeting LRP1 presents a promising strategy for the development of cancer immunotherapies. However, to date, the regulation mechanism of LRP1 in tumors remains controversial. For instance, SRE binding protein (SREBP) and GR element (GRE) modulate LRP1 mRNA levels,^[^ ^13^, ^57^ ^]^ while activated insulin signaling can promote LRP1 degradation via the proteasome system.^[^ ^58^ ^]^ Other studies suggest that aggregated LDL prolongs LRP1 half‐life by preventing its ubiquitination through CHFR inhibition.^[^ ^59^ ^]^ In our study, we discovered that LMO7 deficiency significantly upregulates LRP1 expression in macrophages. Moreover, we identified LMO7 as an E3 ubiquitin ligase that mediates K48‐linked polyubiquitination at lysine 45 (K45) of the LRP1 β chain, promoting its degradation via the proteasome and thereby suppressing macrophage phagocytosis. These findings not only reveal a novel post‐translational regulatory mechanism of LRP1 but also underscore the immunomodulatory potential of targeting the LMO7‐LRP1 axis in TAMs.

Macrophage‐based immunotherapies that harness phagocytosis have recently gained momentum due to their dual role in eliminating tumor cells and stimulating adaptive immunity. In addition to antibody‐dependent cellular phagocytosis induced by rituximab or cetuximab,^[^ ^60^, ^61^ ^]^ recent strategies have focused on targeting phagocytosis checkpoints to enhance macrophage‐mediated clearance. Nowadays, over 23 CD47 targeting therapeutic agents are under investigation across 46 clinical trials.^[^ ^4^ ^]^ Monoclonal antibodies such as BND‐22, Pembrolizumab, and IMM47 were also designed to interfere with the interaction between phagocytosis checkpoint signal to enhance the engulfment of cancer cells by phagocytes.^[^ ^62^, ^63^, ^64^ ^]^ However, these agents frequently induce hematological toxicities, limiting their clinical utility.^[^ ^65^ ^]^ Another promising strategy involves amplifying “eat me” signals on tumor cells, such as calreticulin exposure, through chemotherapy, radiotherapy, or immunotherapy.^[^ ^66^ ^]^ Enhancing macrophage recognition of these signals using antibodies or immunomodulators is an active area of drug development. Combination therapies have also shown potential to improve phagocytosis. Previous reports showed that combining a CD47 blocking antibody with Temozolomide, a DNA alkylating agent used in the treatment of glioblastoma, can enhance CD47 antibody‐mediated phagocytosis and lead to tumor regression.^[^ ^67^ ^]^ There have been previous reports regarding the combining enhancement of “eat‐me” signals with blockade of “don't‐eat‐me” signals can temper TNF‐α‐dependent inflammation, and limit atherosclerosis.^[^ ^68^ ^]^ In our study, we reveal that macrophage‐specific LMO7 ablation potentiates anti‐SIRPα therapy. Moreover, to establish a translatable pharmacological approach, leveraging an optimized AAV‐based macrophage‐targeting platform,^[^ ^69^, ^70^ ^]^ we demonstrated that AAV‐mediated LMO7 silencing phenocopied genetic knockout, achieving comparable synergistic tumor suppression with anti‐SIRPα. These results highlight the potential of integrating LMO7‐targeted therapies with existing checkpoint inhibitors to optimize macrophage‐based immunotherapy. In the future, beyond transcriptional/translational suppression of LMO7, its characterized E3 ubiquitin ligase activity presents an alternative druggable vulnerability. Structure‐guided development of small‐molecule inhibitors targeting the F‐box ubiquitin ligase domain could yield pharmacologically tractable agents for combination immunotherapy regimens.

## 4 Conclusion

In conclusion, this study identifies LMO7 as a critical intrinsic regulator of TAM function and tumor immunity. By promoting the ubiquitination and degradation of LRP1, LMO7 impairs macrophage phagocytosis and contributes to tumor immune evasion. Targeting LMO7, particularly in combination with anti‐SIRPα therapy, offers a novel approach to improve macrophage‐targeted cancer immunotherapy and enhance patient outcomes.

## 5 Experimental Section

### Mice

Genomic LMO7 knockout mice (*Lmo7* ^−/−^) and conditional LMO7 knockout mice (*Lmo7^fl/fl^*) in the C57BL/6 background were generated by Cyagen Biosciences Inc. (Suzhou, China) as previously reported.^[^ ^28^, ^29^ ^]^ Myeloid‐specific LMO7‐deficient mice (Lyz2^cre^ *Lmo7^fl/fl^*, Lyz2^cre^ *Lmo7^wtl/fl^*) were generated by crossing Lysozyme M‐cre mice (Jackson Laboratory) with *Lmo7^fl/fl^* mice. Mouse genotype identification primers are shown in Table S1 (Supporting Information). All mice were bred and maintained in pathogen‐free conditions under a 12 h light and dark cycle, with stable temperature (20 °C–24 °C) and humidity (45%–65%) at Shanghai Jiaotong University (Shanghai, China). All experimental procedures involving animals were approved by the Animal Care and Use Committee of Shanghai Jiaotong University and conducted in accordance with the “Animal Management Regulations” (revised 2024) issued by the National Science and Technology Commission (A2023219‐011).

### Reagents

D‐Luciferin potassium salt (ST‐196) and DAPI (C1005) were purchased from Beyotime Biotechnology (Shanghai, China). Collagenase D (11088866001), DNase I (10104159001), Triton X‐100 (2315025), BSA (SRE0098), protease inhibitor cocktail (P8340), and PKH26 (PKH26GL) were obtained from Sigma–Aldrich (St. Louis, MO, USA). Antibodies for Flag‐Tag (14793S, RRID: AB_2572291), HA‐Tag (3724S, RRID: AB_1549585), Myc‐Tag (2272S, RRID: AB_10692100), LRP‐1 (64099S, RRID: AB_2799654), F4/80 (30325S, RRID: AB_2798990), CD4 (25229S, RRID: AB_2798898), CD8 (98941S, RRID: AB_2756376), and β‐actin (8457S, RRID: AB_10950489) were sourced from Cell Signaling Technology (Boston, MA, USA). The Alexa Fluor 488‐conjugated Goat anti‐rabbit secondary antibody (111‐545‐003, RRID: AB_2338046) and Alexa Fluor 594‐conjugated Goat anti‐mouse secondary antibody (115‐585‐003, RRID: AB_2338871) were purchased from Jackson ImmunoResearch (West Grove, PA, USA). The Fixable Viability Stain 510 (564406, RRID: AB_2869572), APC‐Cy7 conjugated CD45 (557659, RRID: AB_396774), PE‐Cy7 conjugated Ly6C (560593, RRID: AB_1727557), BV650 conjugated Ly6G (740554, RRID: AB_2740255), FITC conjugated CD11b (561688, RRID: AB_10898180), BV605 conjugated F4/80 (743281, RRID: AB_2741399), AF647 conjugated I‐A/I‐E (562367, RRID: AB_11152078), PE conjugated CD206 (568273, RRID: AB_2916867), BV786 conjugated IFN‐γ (563773, RRID: AB_2738419), FITC‐conjugated CD3 (553061, RRID: AB_394594), BUV737 conjugated CD4 (612761, RRID: AB_2870092), PE‐CF594 conjugated CD8a (562315, RRID: AB_11154579) and BV421 conjugated FOXP3 (562996, RRID: AB_2737940) were purchased from BD Bioscience (San Jose, CA, USA). The anti‐human CD47 antibody (GM‐27657AB) was sourced from Genomeditech (Shanghai, China). The anti‐mouse CD47 antibody (MIAP301, RRID: AB_627088) was purchased from Santa Cruz Biotechnology (Dallas, USA). Antibodies for CD3ε (GM‐87887MAB) and SIRPα (GM‐87905MAB) for in vivo experiments were purchased from Genomeditech (Shanghai, China). AAV9‐F4/80‐Empty‐EF1‐ZsGreen1‐WPRE (101216GP‐AAV), AAV9‐F4/80‐Cre‐EF1‐ZsGreen1‐WPRE (101205GO‐AAV), AAV9‐F4/80‐eGFP‐5′miR30‐*Scramble‐*shRNA‐3′miR30‐WPRE (105801GY‐AAV) and AAV9‐F4/80‐eGFP‐5′miR30‐*Lmo7‐*shRNA‐3′miR30‐WPRE (113563GM‐AAV) (Mouse *Lmo7*‐shRNA sequence: 5′‐GATCCGCTATTAACAACACCAAGTTTTTCAAGAGAAAACTTGGTGTTGTTAATAGCTTTTTTG‐3′)^[^ ^28^ ^]^ were obtained from Genomeditech (Shanghai, China). RPMI‐1640 medium (11875093), DMEM medium (11965092), fetal bovine serum (FBS, 10099), and penicillin–streptomycin (15070063) were obtained from Gibco (Grand Island, NY, USA). Calcein‐AM (40719ES60), PEI transfection reagent (40816E), plasmid extraction mini kits (19001ES70), Hifair AdvanceFast One‐step RT‐gDNA Digestion SuperMix for qPCR (11151ES60), and Hieff UNICON Universal Blue qPCR SYBR Green Master Mix (11184ES08) were sourced from Yeasen Biotech (Shanghai, China). LRPAP1 protein was purchased from MedChemExpress (New Jersey, USA). The KOD‐Plus‐Mutagenesis Kits (SMK‐101) were obtained from TOYOBO (Osaka, Japan). The Protein A/G Agarose beads (SA032100) were obtained from Smart‐Lifescience (Changzhou, China). The nitrocellulose membranes (10600002) were sourced from GE Healthcare (Little Chalfont, UK). The PE conjugated granzyme B (12‐8898‐80, RRID: AB_10853811), ECL detection kits (A38555), and Trizol (15596018CN) were purchased from Thermo Fisher Scientific (Waltham, MA, USA).

### Cell Culture

The mouse colon cancer cell line MC38 (RRID: CVCL_B288), the human embryonic kidney cell line HEK293T cells (RRID: CVCL_0063), and the human cervical carcinoma HeLa cell line (RRID: CVCL_0030) were cultured in DMEM or RPMI‐1640 (Gibco), supplemented with 10% fetal bovine serum (FBS, Gibco) and 1% penicillin‐streptomycin (Yeasen Biotech). All cell lines were obtained from ATCC and tested negative for mycoplasma contamination. The MC38‐luciferase cells were obtained by infecting normal MC38 cells with CMV‐Luc‐Puro Lentivirus (GM‐0220IV210, Genomeditech) and selecting with puromycin. Bone marrow‐derived macrophages (BMDMs) were isolated from wild type or LMO7 knockout C57BL/6 mice as previously described.^[^ ^71^ ^]^ Briefly, bone marrow cells were extracted from the femurs and tibias and cultured in DMEM (Gibco) supplemented with 10 ng mL^−1^ recombinant mouse M‐CSF for 5–7 days. All cells were maintained at 37 °C in a humidified incubator with 5% CO_2_.

### Animal Models

MC38 cells (5 × 10^5^) were inoculated subcutaneously into 6–8 weeks female *Lmo7* ^−/−^, Lyz2^cre^ *Lmo7^wt/fl^*, Lyz2^cre^ *Lmo7^fl/fl^* mice, and their corresponding control mice. Tumor size was measured every 2 days using calipers equipped with a vernier scale, and tumor volume was calculated using the formula (length × width^2^)/2. At the conclusion of the experiment, mice were sacrificed, and tumors were harvested for analysis via FACS, single‐cell RNA‐seq or immunofluorescence staining. For anti‐CD3ε antibody treatment experiments, mice were randomized into treatment groups, tumor‐bearing mice were administered anti‐CD3ε antibody (10 mg kg^−1^) via intraperitoneal injection every 3 days starting from day ‐1 for a total of five doses. For AAV9‐F4/80‐Cre or AAV9‐F4/80‐*Lmo7*‐shRNA treatment experiments,^[^ ^70^, ^72^ ^]^ mice were randomized into treatment groups, tumor‐bearing mice were injected via the tail vein with 100 µL of AAV (2 × 10^11^ viral genomes) on the same day with tumor cell inoculation.^[^ ^69^, ^73^ ^]^ For LRPAP1 treatment experiments, mice were randomized into treatment groups, and tumor‐bearing mice received intratumoral injections of LRPAP1 (0.2 mg kg^−1^) every 2 days starting from day 6 for a total of four doses. For anti‐SIRPα antibody treatment experiments, mice were randomized into treatment groups, tumor‐bearing mice were administered anti‐SIRPα antibody (20 mg kg^−1^) via intraperitoneal injection every 2 days starting from day 6 for a total of four doses. At the end of the experiment, mice were sacrificed, and tumors were collected and analyzed by flow cytometry. For in vivo imaging, MC38‐luciferase cells (5 × 10^5^) were inoculated subcutaneously into Lyz2^cre^ *Lmo7^fl/fl^* mice and their corresponding control. Mice were given luciferin (150 mg kg^−1^) by intraperitoneal injection and imaged using the IVIS Lumina Series III (PerkinElmer, USA).

### Single‐Cell RNA‐Seq

Approximately 10000 FACS‐isolated live cells (1000 cells µL^−1^ suspension) were resuspended in 1x PBS (calcium‐ and magnesium‐free) containing 0.04% BSA (A2153, SIGMA). The cell suspension was used as input in 10× Chromium Controller System (10× Genomics Inc., product code 120223), and cell barcoding was performed using gel beads in emulsion (GEMs) in assembly chip. GEM reverse transcription (RT) reactions were conducted in a thermocycler at 53 °C for 45 min, followed by 85 °C for 5 min, and held at 4 °C overnight. SPRIselect dynabeads (B23318, Beckman Coulter) were utilized for GEM recovery after RT. DNA quantities ranging from 2 to 50 ng were used for target enrichment. cDNA amplification, fragmentation, end repair, and A‐tailing preparation were performed according to the manufacturer's protocol. Quality control and quantification of the gene expression library were conducted at various stages using high‐sensitivity DNA chips and an Agilent 2100 Bioanalyzer (Agilent Technologies). Quality control was performed twice before sequencing. Two barcoded scRNA samples were pooled together before sequencing, which was performed on the NovaSeq 6000 S1 platform with a sequencing depth of ≈300 million reads.

### scRNA‐Seq Data Processing

The Seurat (version 5.0) R package was employed for comprehensive analysis of the scRNA‐seq data. Low‐quality cells were filtered by applying thresholds for minimum gene expression, mitochondrial gene content, and cell complexity, based on the number of detected genes and total molecular counts. The data were normalized using the LogNormalize function. Highly variable features were identified and used for dimensionality reduction via Principal Component Analysis (PCA). Significant principal components (PCs) were selected based on the elbow plot, and these PCs were used for clustering with the Louvain algorithm. Clusters were visualized using Uniform Manifold Approximation and Projection (UMAP) for dimensionality reduction. Marker genes for each cluster were identified by comparing gene expression profiles between clusters using the Wilcoxon rank‐sum test. Cell types were annotated by referencing the expression of well‐established marker genes and by comparison with publicly available single‐cell datasets. Differentially expressed genes between clusters of interest were identified using the FindMarkers function. CNVs were inferred from scRNA‐seq data using the inferCNV R package (https://github.com/broadinstitute/inferCNV), with default parameters. Raw count matrices were input along with corresponding cell type annotations. Immune cells were used as reference.^[^ ^74^ ^]^

### CellChat Analysis

CellChat v2.1.0 (github.com/jinworks/CellChat) was employed to analyze and visualize cell–cell interactions. A CellChat object was generated by using cell‐type annotations and overexpressed ligand‐receptor interactions identified from the processed scRNA‐seq data. Communication probabilities between cell types were calculated using the computeCommunProb function, and the overall interaction network was visualized with netVisual_circle. To investigate pathway‐specific communication, the computeCommunProbPathway function was used to identify signaling pathways enriched between specific cell types, and netVisual_aggregate was applied to visualize these pathway interactions. Default parameters were used unless stated otherwise.

### Flow Cytometry Assay and Cell Sorting

Single‐cell suspensions were prepared from fresh mouse tumor tissues using the Tumor Dissociation Kit, mouse (MACS, 130‐0960‐730), with the Miltenyi Biotec gentleMACS Octo Dissociator with Heaters, following the 37C‐m‐TDK‐1 program (Miltenyi Biotec), and passing through 70 µm cell strainers.^[^ ^75^ ^]^ After red blood cell (RBC) lysis, cells were preincubated with Fixable Viability Stain 510 (BD Horizon, 564406) to detect dead cells and Fc blocking anti‐CD16/32 antibody (BD Pharmingen, 553141) to block non‐specific FcγR binding prior to staining with primary antibodies. Cells were incubated with the specific flow antibodies for 40 min at 4 °C for surface staining. For nuclear transcription factor staining, cells were surface‐stained, fixed, and permeabilized using Transcription Factor Buffer Set (BD Pharmingen, 562574), followed by staining with anti‐mouse CD206 or FOXP3 antibodies. All samples were analyzed via flow cytometry (LSRFortessa X‐20, BD Bioscience), and the data were processed using FlowJo software (RRID: SCR_008520).

For tumor infiltrated macrophage sorting, single‐cell suspensions from fresh tumors were prepared as previously described, then stained with anti‐mouse CD45, anti‐mouse CD11b, anti‐mouse Ly6G antibodies, anti‐mouse Ly6C antibodies, and anti‐mouse F4/80 antibodies. Tumor infiltrated macrophages were sorted using a BD FACSAria III flow cytometer.

Each cell lineage was identified as follows: tumor associated macrophages (TAMs): CD45^+^ CD11b^+^ Ly6G^−^ Ly6C^−^ F4/80^+^; MHC‐II^+^ macrophages: CD45^+^ CD11b^+^ Ly6G^−^ Ly6C^−^ F4/80^+^ MHC‐II^+^; CD206^+^ macrophages: CD45^+^ CD11b^+^ Ly6G^−^ Ly6C^−^ F4/80^+^ CD206^+^; M‐MDSCs: CD45^+^ CD11b^+^ Ly6G^−^ Ly6C^+^; PMN‐MDSCs: CD45^+^ CD11b^+^ Ly6G^+^ Ly6C^low^; CD8^+^ T cells: CD45^+^ CD3^+^ CD8^+^ CD4^−^; CD4^+^ T cells: CD45^+^ CD3^+^ CD8^−^ CD4^+^; Regulatory T cells: CD45^+^ CD3^+^ CD8^−^ CD4^+^ FOXP3^+^.

### RNA Extraction and qRT‐PCR Analysis

Total RNA was extracted using TRIzol (Invitrogen) reagent and reverse transcribed into cDNA with the Hifair AdvanceFast One‐step RT‐gDNA Digestion SuperMix for qPCR (Yeasen). The analyzed genes were provided in Table S2 (Supporting Information). The relative expression levels of target genes, normalized to the housekeeping gene Gapdh, were quantified by real‐time quantitative PCR (qPCR) using Hieff UNICON Universal Blue qPCR SYBR Green Master Mix (Yeasen) on a Bio‐Rad CFX96 real‐time system (Bio‐Rad, USA). The amplification protocol involved an initial denaturation at 95 °C for 10 min, followed by 40 cycles of 15 s at 95 °C and 1 min at 60 °C. The fold change in gene expression was calculated using the 2^(‐ΔΔCt) method.

### Phagocytosis Assay

For the in vitro phagocytosis assay, BMDMs were trypsinized with 0.25% trypsin, then centrifuged and resuspended in FBS‐free DMEM. Each reaction contained 0.1 million BMDMs in 200 µL DMEM, incubated at 37 °C. Cancer cells were labeled with Calcein‐AM (Yeasen) per the manufacturer's instructions, washed, and resuspended in FBS‐free DMEM. Aliquots (0.2 million cells in 200 µL DMEM) were added to BMDMs, followed by anti‐CD47 antibody treatment and incubation for 4 h at 37 °C. After co‐culture, cells were stained with anti‐mouse F4/80 antibodies, incubated at 4 °C for 40 min, and analyzed by flow cytometry (LSRFortessa TM X‐20, BD Bioscience). The phagocytosis rate was quantified as the percentage of F4/80^+^ Calcein‐AM^+^ cells relative to total F4/80^+^ cells. For immunofluorescence‐based detection, BMDMs were labeled with PKH26 (Sigma‐Aldrich), and cancer cells were prepared as described. After 4 h of co‐culture, cells were seeded onto glass slides, fixed with 4% paraformaldehyde, and imaged using a Leica TCS SP8 confocal microscope.

For the *ex vivo* phagocytosis assay, tumor infiltrated macrophages were isolated from fresh tumors via flow cytometry (BD FACSAria III) and co‐cultured with cancer cells following the in vitro protocol. Phagocytosis was assessed by flow cytometry. For in vivo phagocytosis assays with PMs, cancer cells were pre‐incubated with anti‐CD47 antibodies for 2 h, labeled as previously described, and injected intraperitoneally. After 4 h, peritoneal lavage fluid was collected, and cells were stained with anti‐mouse F4/80 antibodies before flow cytometry analysis. Phagocytosis was quantified as the percentage of F4/80⁺ Calcein‐AM⁺ cells among total F4/80⁺ cells. For immunofluorescence analysis, F4/80⁺ PMs were isolated by FACS, seeded onto glass slides, fixed with 4% paraformaldehyde, stained with DAPI, and imaged using a Leica TCS SP8 confocal microscope. For in vivo phagocytosis assays with tumor‐infiltrating macrophages, PKH26‐labeled cancer cells were injected subcutaneously. Upon reaching a specified tumor size, tumors were excised, dissociated into single‐cell suspensions, and analyzed by flow cytometry. Tumor‐infiltrating macrophages were identified as CD45⁺ CD11b⁺ Ly6G^−^ Ly6C^−^ F4/80⁺, and phagocytosis was quantified as the percentage of CD45⁺ CD11b⁺ Ly6G^−^ Ly6C^−^F4/80⁺ PKH26⁺ cells among total CD45⁺ CD11b⁺ Ly6G^−^ Ly6C^−^ F4/80⁺ cells. For immunofluorescence analysis, CD45⁺ CD11b⁺ Ly6G^−^ Ly6C^−^ F4/80⁺ macrophages were isolated by FACS, seeded onto glass slides, fixed with 4% paraformaldehyde, stained with DAPI, and imaged using a Leica TCS SP8 confocal microscope.

### Plasmid Construction

The cDNA sequences of LMO7 and the LRP1 β chain were synthesized by HUAGENEBIO and cloned into the pcDNA3.1‐Myc or pcDNA3.1‐Flag eukaryotic expression vectors, respectively. The plasmid encoding ubiquitin (Ub) was generously provided by Prof. Baoxue Ge (Shanghai Tongji University, China). Plasmids for Ub variants (Ub‐K6, Ub‐K11, Ub‐K27, Ub‐K29, Ub‐K33, Ub‐K48, Ub‐K63, and Ub‐K48R) were obtained as previously reported. Site‐directed mutant plasmids of the LRP1 β chain (K45R, K143R, K400R, K413R, K509R, K584R) were generated using the KOD‐Plus‐Mutagenesis Kit (Toyobo), following the manufacturer's protocol. Plasmids for all constructs used in transfection experiments were purified using Plasmid Extraction Mini Kits (Yeasen), and the accuracy of all constructs was verified by DNA sequencing.

### Immunoprecipitation and Western Blot Analysis

The assays were performed as previously described.^[^ ^76^ ^]^ Cells were lysed in radioimmunoprecipitation assay (RIPA) buffer (Beyotime) supplemented with a protease inhibitor cocktail (Sigma‐Aldrich). The lysates were centrifuged at 4 °C, and the supernatants were collected as the protein extracts. To reduce nonspecific binding, the protein extracts were pre‐cleared by incubation with Protein A/G Agarose beads (Smart‐Lifescience) for 1 h. Subsequently, the pre‐cleared extracts were incubated overnight with Protein A/G Agarose beads and the specific antibody, with constant rotation at 4 °C. The resulting immune complexes were washed and subjected to immunoblotting. For Western blot analysis, proteins were transferred onto a nitrocellulose membrane (GE Healthcare BioScience), and after incubation with primary and secondary antibodies, the signal was developed using an ECL detection kit (Thermo) and visualized with the ChemiDoc XRS+ system (Bio‐Rad).

### Statistical Analysis

Data are presented as the mean ± SEM, with a sample size of *n* ≥ 3 for all experiments. Detailed statistical parameters of the analyses are provided in the Figure Legends. For two groups, Student's *t*‐test was applied to assess mean values. For multiple groups, one‐way or two‐way ANOVA was performed, followed by Tukey's test or Sidak's test. For survival analysis, log‐rank test was performed. Statistical significance was set at ns, non‐significance, ^*^p < 0.05, ^**^p < 0.01, ^***^p < 0.001. All statistical analyses were conducted using GraphPad Prism version 8 software (GraphPad Software, San Diego, CA, USA, RRID: SCR_002798).

## Conflict of Interest

The authors declare no conflict of interest.

## Author Contributions

M.L. and T.W. contributed equally to this work. L.S. and F.Q. conceived the study. M.L., T.W., L.S., and F.Q. designed the experiments and drafted the manuscript. M.L., Y.Z and Z.W. performed and interpreted experimental data. W.L. and Z.W. provided flow cytometry antibodies and technical support. M.L., X.Y. and Y.Q. constructed and identified the knockout mouse. T.W. and J.X. offered critical advice for manuscript construction. F.Q., L.S., W.L., Z.W. and W.G. provided funding for this study. All authors read and approved the final manuscript.

## Supporting information



Supporting Information

## Data Availability

The authors confirm that all supporting data for this research are either included in the manuscript or can be obtained from the corresponding author upon reasonable request. Our newly generated single‐cell RNA sequencing datasets have been deposited in the Gene Expression Omnibus (GEO) repository under accession code GSE294033. Additionally, this investigation incorporated analysis of existing publicly available scRNA‐seq data (GSE245552) retrieved from the GEO database.
