# Supplementary material for: LMO7 Suppresses Tumor‐Associated Macrophage Phagocytosis of Tumor Cells Through Degradation of LRP1
Source: Adv Sci (Weinh). 2025 Nov 9;13(35):e11162. doi: 10.1002/advs.202511162 (PMC13292253; doi:10.1002/advs.202511162)
Supplement: Supplementary file 1 — Supporting Information [file ADVS-13-e11162-s001.pdf]

Supplementary Figure S1

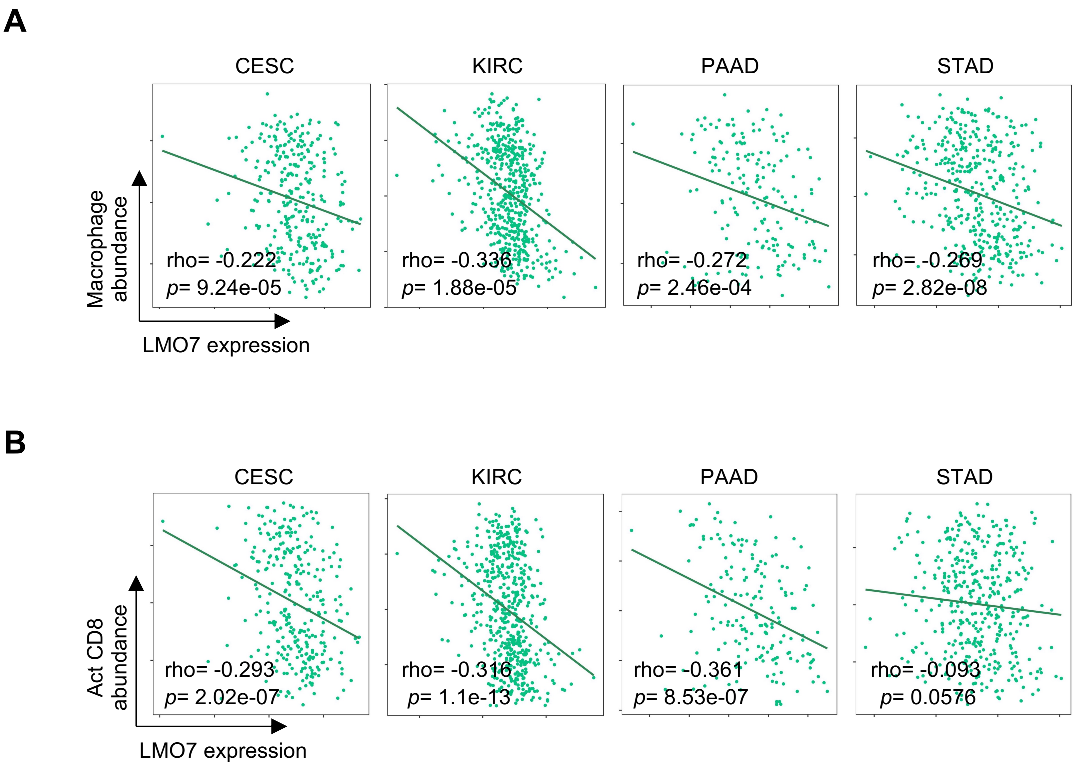

Supplementary Figure S1. The correlation between immune cell abundance and LMO7 expression.

A. The correlation between macrophage abundance and LMO7 expression in CESC, KIRC, PAAD and STAD. **B.** The correlation between activated $CD8^+$ T cell abundance and LMO7 expression in CESC, KIRC, PAAD and STAD.

Supplementary Figure S2

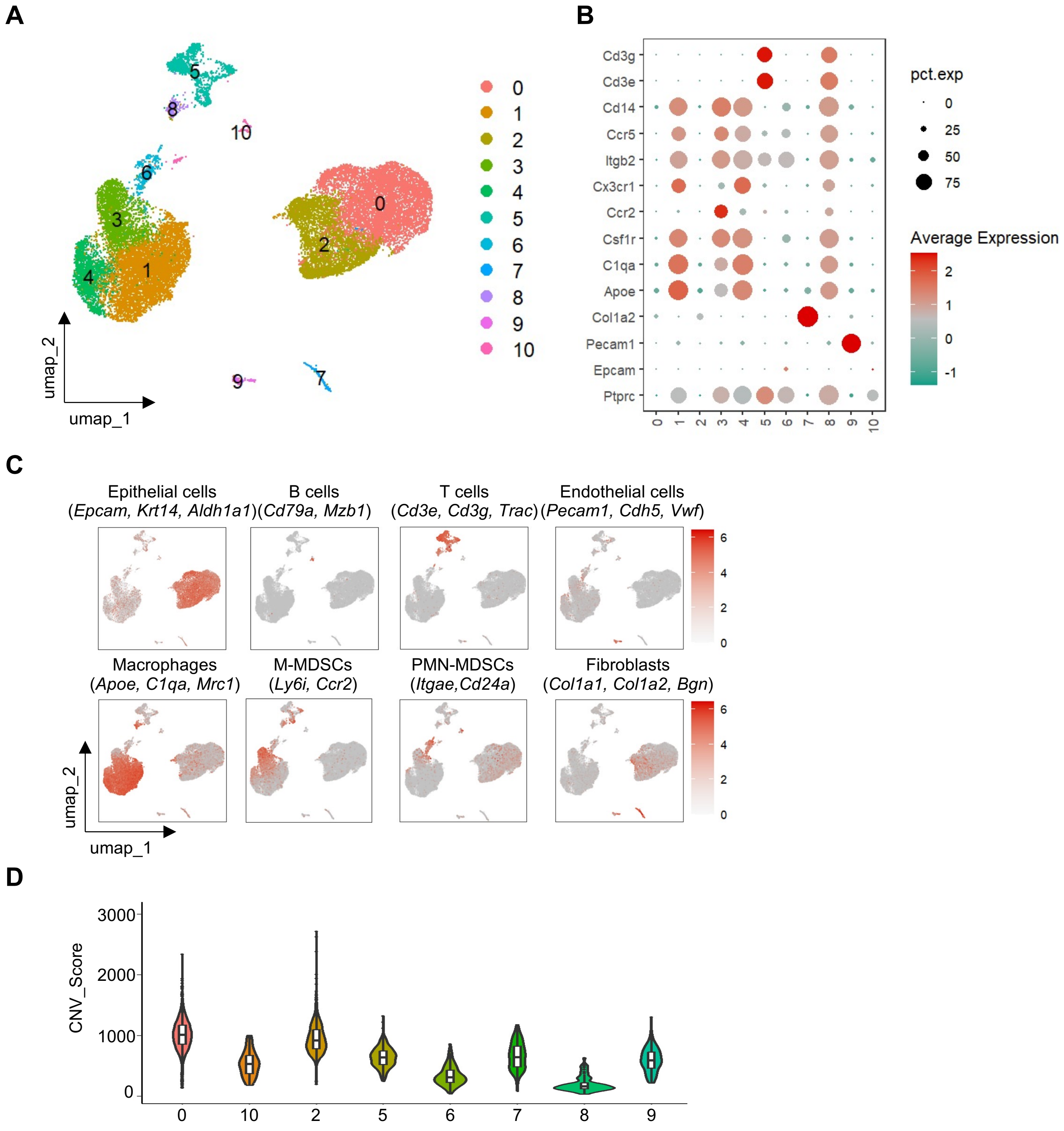

Supplementary Figure S2. Cell annotation of scRNA-seq from mice bearing MC38 tumors.

A. Umap plot showing the results after unbiased clustering. Subpopulation of eleven clusters were identified. **B.** Dot plot presenting the signature genes of the clusters. **C.** Umap plot displaying the expression of feature genes sets in cell types. **D.** Violin plot showing CNV scores across different clusters, using the main immune clusters (Cluster 1, 3, 4) as references.

Supplementary Figure S3

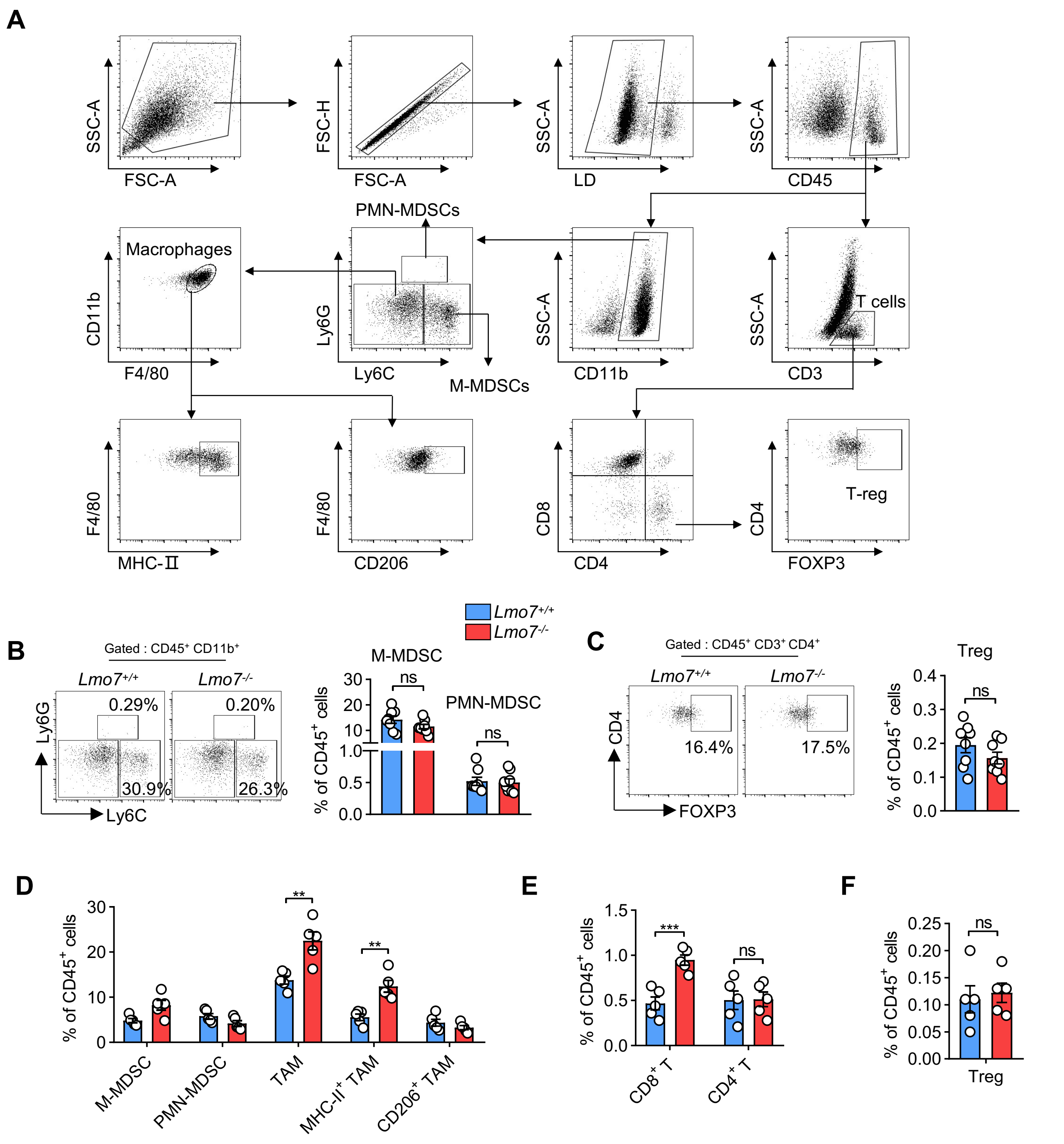

Supplementary Figure S3. FACS analysis of immune cells in MC38 or B16F10 bearing *Lmo7*$^{+/+}$ and *Lmo7*$^{-/-}$ mice.

A. Gating strategy of flow cytometry analysis of immune cells in mouse tumors. FACS analysis and quantification of tumor-infiltrating MDSCs (**B**) and regulatory T cells (**C**) in MC38 tumors. FACS analysis and quantification of tumor-infiltrating MDSCs, macrophages (**D**), T cells (**E**) and regulatory T cells (**F**) in B16F10 tumors. The data are representative of three independent experiments (means ± SEM), n = 8 mice per group (**B**, **C**) or n = 5 mice per group (**D**, **E**, **F**). *p* values were analyzed using unpaired two-tailed *t*-tests (**B**, **C**, **D**, **E**, **F**). ns, non-significance, $^{**}p < 0.01$, $^{***}p < 0.001$.

Supplementary Figure S4

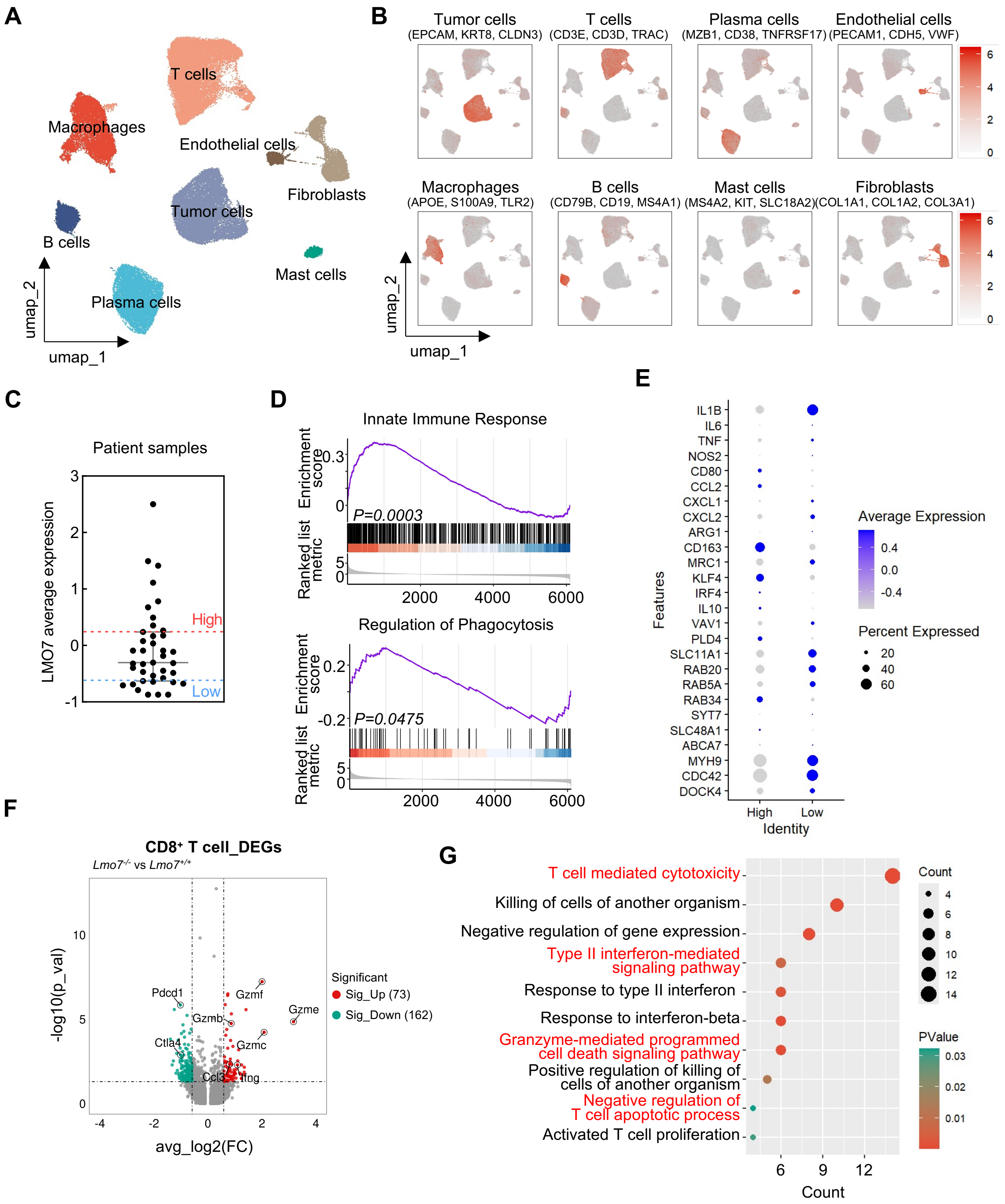

Supplementary Figure S4. scRNA-seq analysis of COAD cancer patients and CD8$^+$ T cells in MC38 bearing *Lmo7*$^{+/+}$ and *Lmo7*$^{-/-}$ mice.

A. Umap plot of scRNA-seq analysis from COAD cancer patients (GSE245552). **B.** Umap plot displaying the expression of feature genes sets in all cell types. **C.** Dot plot presenting the average expression of LMO7 and quartile cutoffs in COAD cancer patients. **D.** GSEA enrichment analysis using the total changed genes in Low-LMO7 and High-LMO7 macrophages. **E.** Relative expression of genes related to macrophage function between Low-LMO7 and High-LMO7 macrophages from COAD cancer patients. **F.** Volcano plot showing the -log10 p_val vs log2 fold-change of genes differentially expressed between CD8$^+$ T cells from MC38 bearing *Lmo7*$^{-/-}$ and *Lmo7*$^{+/+}$ mice. **G.** GOBP enrichment analysis using the up-regulated DEGs of CD8$^+$ T cells.

Supplementary Figure S5

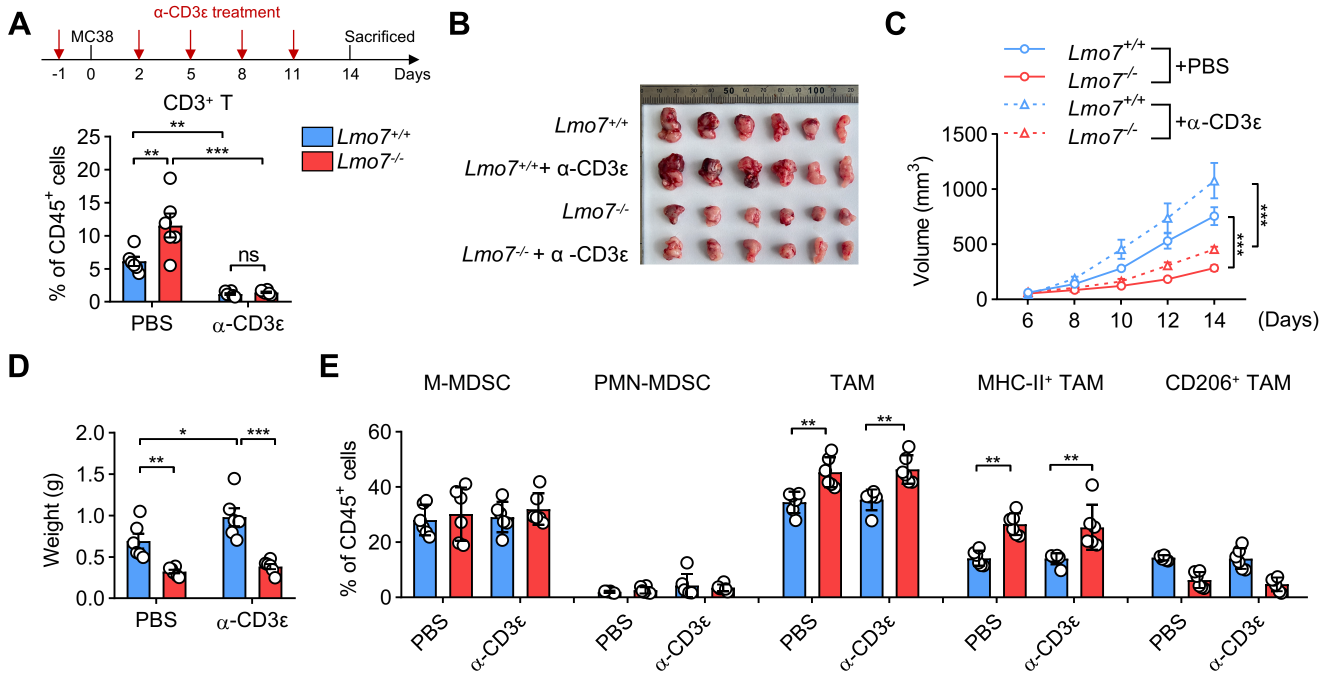

Supplementary Figure S5. LMO7 deficiency enhances immune-mediated tumor confinement not through T cells.

MC38 bearing *Lmo7*$^{+/+}$ and *Lmo7*$^{-/-}$ mice were treated with anti-CD3ε antibody on days -1, 2, 5, 8, 11. **A.** FACS analysis and quantification of tumor-infiltrating CD3^{+} T cells after anti-CD3ε antibody treatment. Tumor photo (**B**), tumor growth curves (**C**), and tumor weight (**D**) of MC38 bearing *Lmo7*$^{+/+}$ and *Lmo7*$^{-/-}$ mice. **E.** FACS analysis and quantification of tumor-infiltrating MDSCs and macrophages in MC38 bearing *Lmo7*$^{+/+}$ and *Lmo7*$^{-/-}$ mice. The data are representative of three independent experiments (means ± SEM), n = 6 mice per group. p values were analyzed using two-way ANOVA (Sidak's test) (**A**, **C**, **D**, **E**). ns, non-significance, *$p < 0.05$, **$p < 0.01$, ***$p < 0.001$.

Supplementary Figure S6

A

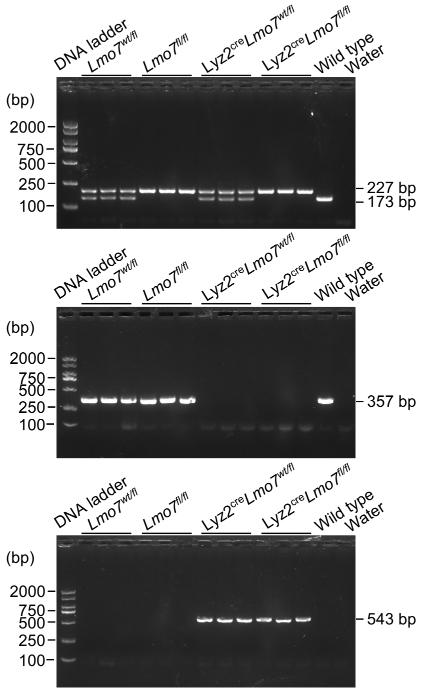

B

Mice Type	WT (*Lmo7* 173 bp)	flox/flox (Neo 227 bp)	$cre^{-/-}$ (WT 357 bp)	$cre^{+/+}$ (cre 543 bp)
Wild Type	+	-	+	-
Lmo7$^{wt/fl}$	+	+	+	-
Lmo7$^{fl/fl}$	-	+	+	-
Lyz2^{cre}*Lmo7*$^{wt/fl}$	+	+	-	+
Lyz2^{cre}*Lmo7*$^{fl/fl}$	-	+	-	+

C

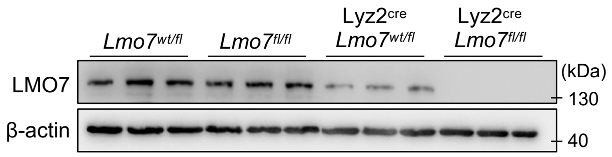

Supplementary Figure S6. Identification of myeloid-specific LMO7-deficient mice.

A. Genotypic identification by PCR analysis of tail tissue DNA. For loxp-targeted allele, homozygous *Lmo7*$^{fl/fl}$ mice have a 227-bp band, wild type mice have a 173-bp, whereas heterozygous *Lmo7*$^{wt/fl}$ mice have both bands. For Lyz2-cre-targeted allele, Lyz2^{cre} mice have a 543-bp band, whereas wild type mice have a 357-bp band. **B.** Expected PCR product sizes for different genotypes. **C.** The expression of LMO7 in BMDMs isolated from *Lmo7*$^{wt/fl}$, *Lmo7*$^{fl/fl}$, Lyz2^{cre}*Lmo7*$^{wt/fl}$ and Lyz2^{cre}*Lmo7*$^{fl/fl}$ mice. The data are representative of three independent experiments, n = 3 mice per group.

Supplementary Figure S7

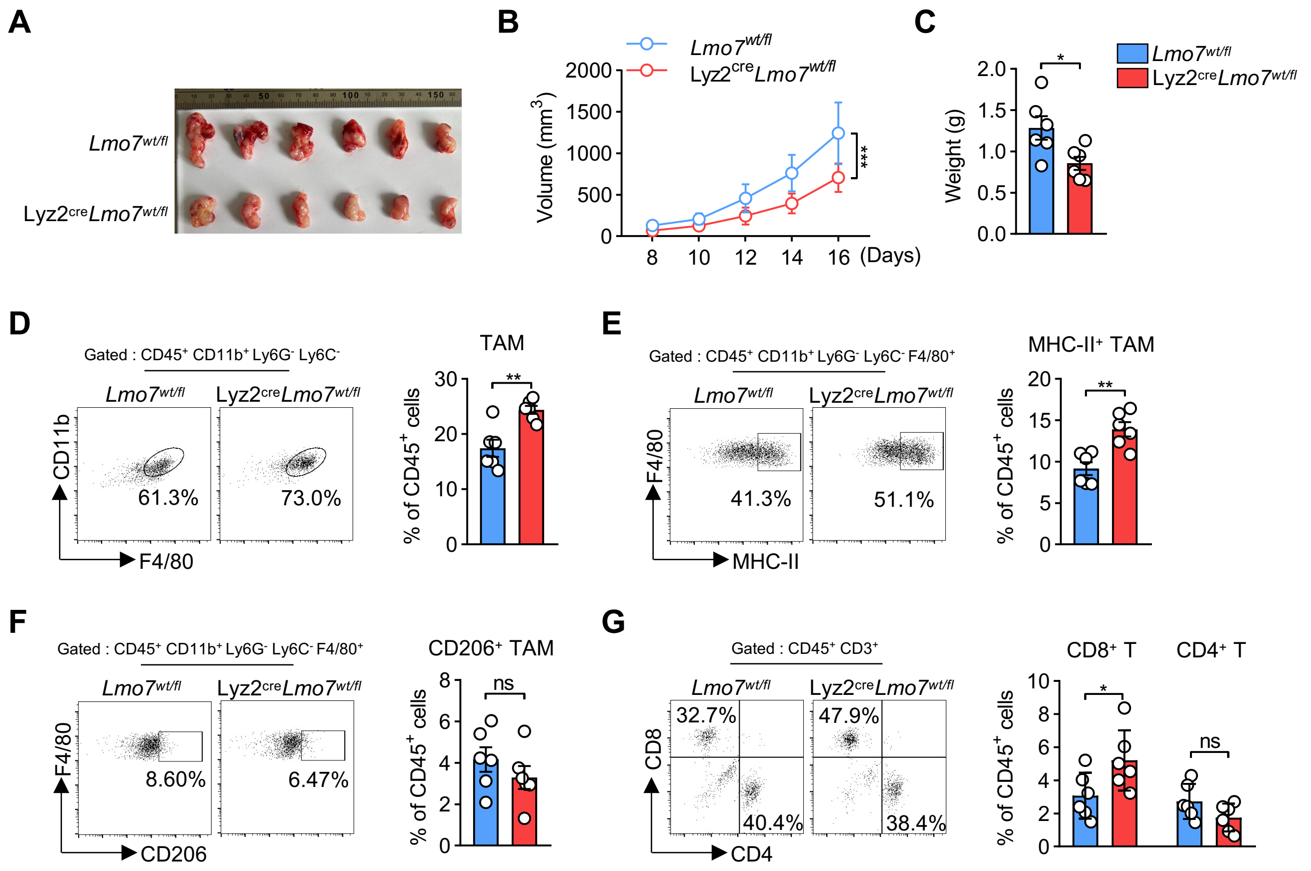

Supplementary Figure S7. Heterozygous LMO7 deletion in macrophages exerts anti-tumor effects.

Tumor photo (**A**), tumor growth curves (**B**), and tumor weight (**C**) of MC38 bearing *Lmo7*$^{wt/fl}$ and Lyz2^{cre}*Lmo7*$^{wt/fl}$ mice. FACS analysis and quantification of TAMs (**D**), MHC-II^{+} TAMs (**E**), CD206^{+} TAMs (**F**) and T cells (**G**) in MC38 tumors. The data are representative of three independent experiments (means ± SEM), n = 6 mice per. *p* values were analyzed using two-way ANOVA (Sidak's test) (**B**), unpaired two-tailed *t*-tests (**C**, **D**, **E**, **F**, **G**). ns, non-significance, *p < 0.05, **p < 0.01, ***p < 0.001.

Supplementary Figure S8

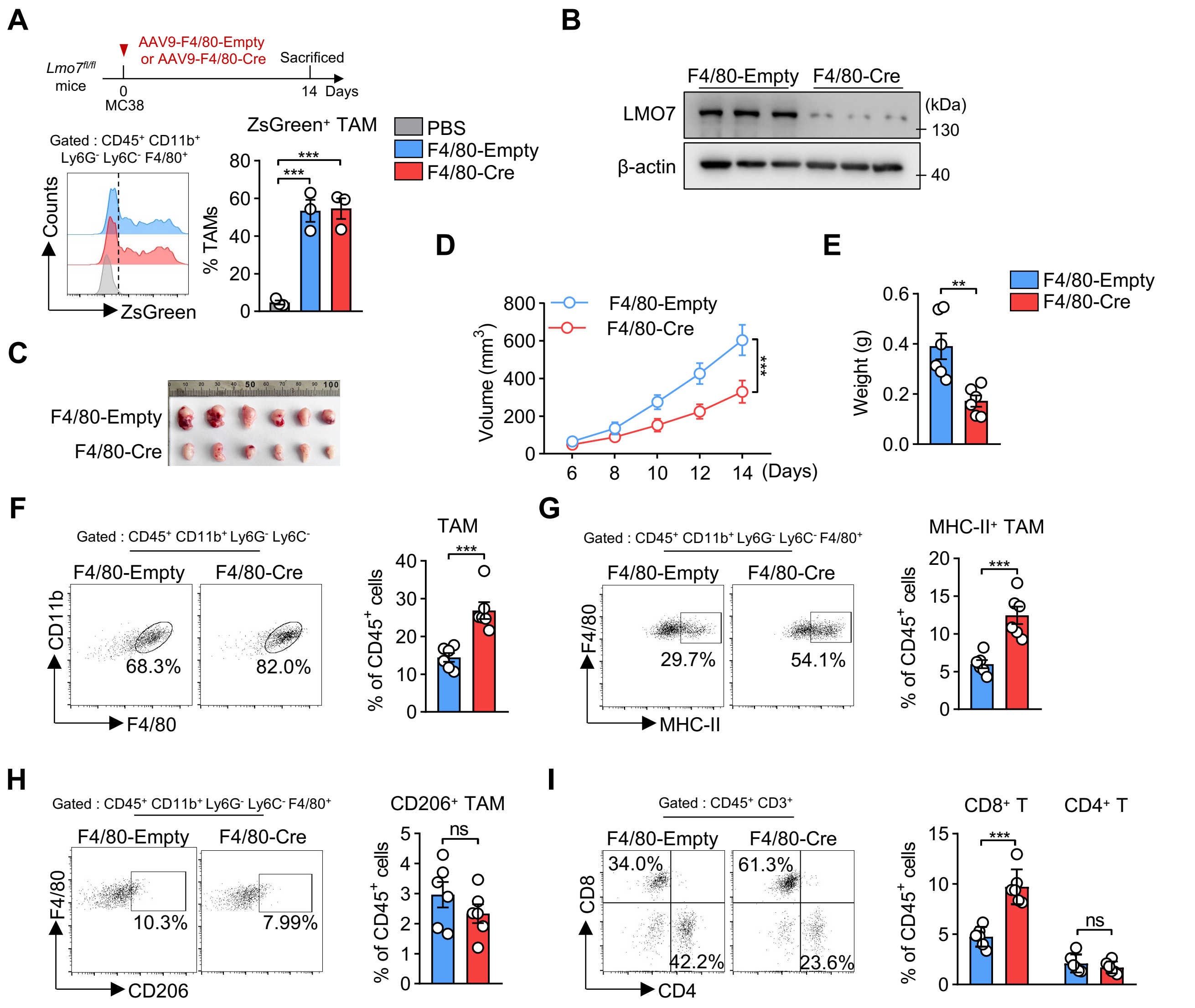

Supplementary Figure S8. AAV9-F4/80-Cre-mediated LMO7 deficiency elicited immune-mediated tumor restriction.

MC38 bearing *Lmo7*$^{fl/fl}$ mice were treated with AAV9-F4/80-Empty or AAV9-F4/80-Cre virus via tail vein on day 0. **A.** FACS analysis and quantification of the ZsGreen fluorescence ratio of TAMs. **B.** The expression of LMO7 in FACS-isolated TAMs. Tumor photo (**C**), tumor growth curves (**D**), and tumor weight (**E**) of MC38 bearing *Lmo7*$^{fl/fl}$ mice. FACS analysis and quantification of TAMs (**F**), MHC-II$^+$ TAMs (**G**), CD206$^+$ TAMs (**H**) and T cells (**I**) in MC38 tumors. The data are representative of three independent experiments (means ± SEM), n = 3 mice per group (**A**, **B**) or n = 6 mice per group (**C**, **D**, **E**, **F**, **G**, **H**, **I**). *p* values were analyzed using one-way ANOVA (Tukey's test) (**A**), two-way ANOVA (Sidak's test) (**D**), unpaired two-tailed *t*-tests (**E**, **F**, **G**, **H**, **I**). ns, non-significance, $^{**}p < 0.01$, $^{***}p < 0.001$.

Supplementary Figure S9

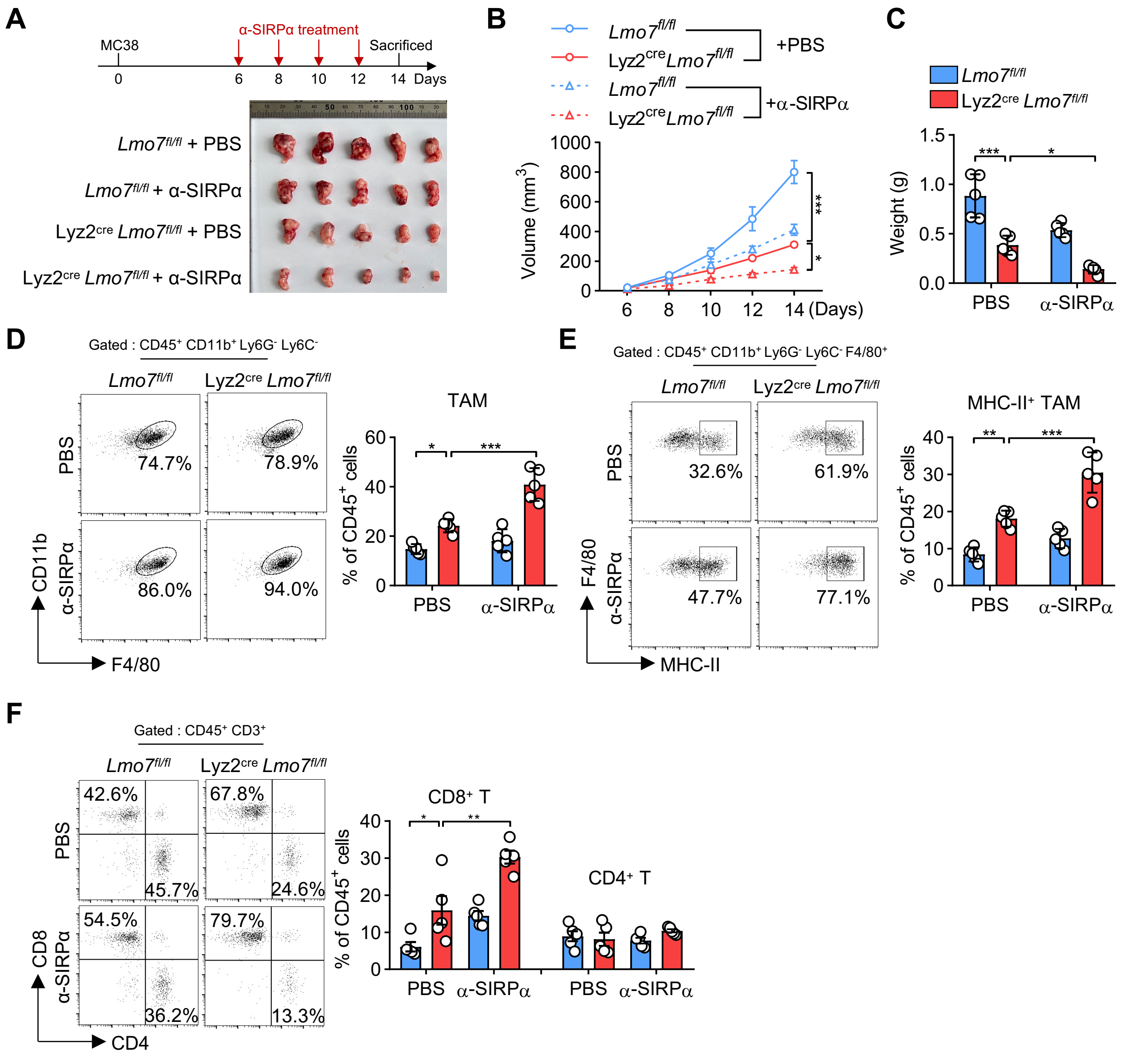

Supplementary Figure S9. LMO7 deficiency combined with anti-SIRPα antibodies promotes TAM-mediated tumor restriction.

Lmo7$^{fl/fl}$ and Lyz2^{cre}*Lmo7*$^{fl/fl}$ mice were treated with or without anti-SIRPα antibody on days 6, 8, 10, 12. Tumor photo (**A**), tumor growth curves (**B**) and tumor weight (**C**) of MC38 bearing *Lmo7*$^{fl/fl}$ and Lyz2^{cre}*Lmo7*$^{fl/fl}$ mice. FACS analysis and quantification of TAMs (**D**), MHC-II^{+} TAMs (**E**) and T cells (**F**) in MC38 tumors. The data are representative of three independent experiments (means ± SEM), n = 5 mice per group. *p* values were analyzed using two-way ANOVA (Sidak's test) (**B**, **C**, **D**, **E**, **F**). *$p < 0.05$, **$p < 0.01$, ***$p < 0.001$.

Supplementary Table S1. Primer pairs for mouse genotyping

Target	Forward (5'-3')	Reverse (5'-3')
Lyz2-cre$^{+/+}$	AGTGCTGAAGTCCATAGATCGG	CTGATTCTCCTCATCACCAGG
Lyz2-cre$^{-/-}$	AGTGCTGAAGTCCATAGATCGG	GTCACTCACTGCTCCCCTGT
Lmo7$^{fl/fl}$	CTGCTTCTGCTGCTACTTTAACCT	TAGCACGTTGATTTCTACCGACT

Supplementary Table S2. Primer pairs for qRT-PCR

Target	Forward (5'-3')	Reverse (5'-3')
Gapdh	CATCACTGCCACCCAGAAGACTG	ATGCCAGTGAGCTTCCCGTTCAG
Vav1	ACAGTGCGTGAACGAGGTCAAG	GCCATAGTTAGCCAGAGACTGG
Pld4	GGAGTCAATGACTCGTCTTCTCG	CAAGACCTGGAGGTCAGTGGAT
Aif1	TCTGCCGTCCAAACTTGAAGCC	CTCTTCAGCTCTAGGTGGGTCT
Dok3	GCCAAGATGACATCCAACTGAGG	CCAGCCTCAAACGAGAACACAC
Tyrobp	GTGACTTGGTGTTGACTCTGCTG	GATAAGGCGACTCAGTCTCAGC
Marco	ATGGCACCAAGGGAGACAAAGG	GCCTGGTTTTCCAGCATCACCT
Rab7	GAGCGGACTTTCTGACCAAGGA	CAATCTGCACCTCTGTAGAAGGC
Rab10	GGACGATGCCTTCAATACCACC	GTGATGGTGTGAAATCGCTCCTG
Rab20	ACCTGAAGCAGTGGCGTTCCTT	CTCCAGCTCAAACAGGCTCTGT
Slc11a1	TGTGTTGGTGGCTGTCTTCCGA	CAAAATGGGCAGTACAGCGAAGG
Slc48a1	GGTTAGGATGCCCACAGACTCA	CACATCCTGGTCAACTTGGAGC
Il1b	TGGACCTTCCAGGATGAGGACA	GTTCATCTCGGAGCCTGTAGGTAGGT
Il6	TACCACTTCACAAGTCGGAGGC	CTGCAAGTGCATCATCGTTGTTC
Tnf	GGTGCCTATGTCTCAGCCTCTT	GCCATAGAACTGATGAGAGGGAG
Nos2	GAGACAGGGAAGTCTGAAGCAC	CCAGCAGTAGTTGCTCCTCTTC
Cd80	CCTCAAGTTTCCATGTCCAAGGC	GAGGAGAGTTGTAACGGCAAGG
Cd86	ACGTATTGGAAGGAGATTACAGCT	TCTGTCAGCGTTACTATCCCGC
Ifnb1	GCCTTTGCCATCCAAGAGATGC	ACACTGTCTGCTGGTGGAGTTC
Ifng	CAGCAACAGCAAGGCGAAAAAGG	TTTCCGCTTCCTGAGGCTGGAT
Cxcl1	TCCAGAGCTTGAAGGTGTTGCC	AACCAAGGGAGCTTCAGGGTCA
Cxcl2	CATCCAGAGCTTGAGTGTGACG	GGCTTCAGGGTCAAGGCAAACT
Cxcl9	CCTAGTGATAAGGAATGCACGATG	CTAGGCAGGTTTGATCTCCGTTC
Cxcl10	ATCATCCCTGCGAGCCTATCCT	GACCTTTTGGCTAAACGCTTTC
Ccl2	GCTACAAGAGGATCACCAGCAG	GTCTGGACCCATTCCTTCTTGG
Ccl5	CCTGCTGCTTTGCCTACCTCTC	ACACACTTGGCGGTTCCTTCGA
